# Estradiol-Dependent Memory and Hippocampal Spine Remodeling Require Proteasomal Protein Degradation

**DOI:** 10.1523/JNEUROSCI.0619-26.2026

**Published:** 2026-10-09

**Authors:** Sarah B. Beamish, Kellie S. Gross, Rachel K. Kuehn, Kailey A. Frank, Elise P. Valentine, Timothy J. Jarome, Karyn M. Frick

**Affiliations:** ^1^Department of Psychological and Brain Sciences, University of Wisconsin-Milwaukee, Milwaukee, Wisconsin 53211; ^2^School of Animal Sciences, School of Neuroscience, Virginia Polytechnic Institute and State University, Blacksburg, Virginia 24061

**Keywords:** dendritic spines, dorsal hippocampus, estrogen, spine morphology, ubiquitin proteasome system

## Abstract

Although 17β-estradiol (E_2_) is a critical neuromodulator of hippocampus-dependent memory and synaptic plasticity, the molecular mechanisms underlying these effects remain incompletely understood. Previous studies showed that E_2_ enhances dorsal hippocampus (DH)-dependent memory consolidation by activating signaling pathways that promote protein synthesis and dendritic spine formation. However, these studies largely overlooked the contribution of protein degradation mediated by the ubiquitin proteasome system (UPS), despite extensive evidence implicating UPS activity in long-term memory and synaptic plasticity. Here, we combined temporally specific dorsal hippocampal infusions of the proteasome inhibitor β-lactone with three-dimensional spine morphometry to examine the role of UPS-mediated protein degradation in E_2_-induced memory consolidation and CA1 spinogenesis. Immediate post-training bilateral infusion of β-lactone into the DH of ovariectomized female mice blocked E_2_-induced enhancement of object recognition memory and attenuated E_2_-associated improvements in spatial memory. Arbor-wide Golgi-based analyses revealed robust E_2_-induced increases in spine number across the apical dendritic tree and smaller parallel effects within the basal arbor. Segment-level analyses showed that E_2_ produced the greatest structural changes on tertiary apical dendritic segments, increasing total, mushroom, and thin spine density and enhancing multiple morphometric features, including mushroom spine surface area and head-to-neck ratio. Secondary basal dendritic segments exhibited smaller, more variable changes. Many E_2_-associated structural effects were attenuated or abolished by β-lactone. Together, these findings provide the first evidence that proteasomal protein degradation contributes to estrogenic enhancement of hippocampus-dependent memory and CA1 dendritic spine remodeling, identifying UPS-mediated protein degradation as a previously unrecognized mechanism regulating estrogenic memory consolidation and structural plasticity.

## Significance Statement

17β-Estradiol (E_2_) rapidly enhances hippocampal memory consolidation and promotes CA1 dendritic spinogenesis, yet the molecular mechanisms that support these effects remain unclear. Here, we combine temporally specific dorsal hippocampal proteasome inhibition with three-dimensional Golgi-based spine analyses to show that proteasomal protein degradation is required for E_2_-induced enhancement of object recognition memory and contributes to E_2_-associated improvements in spatial memory in ovariectomized female mice. We further demonstrate that proteasome activity contributes to E_2_-induced increases in apical CA1 spine density and morphology. These findings reveal new insights into estrogenic modulation of cognition that may provide novel protein targets whose levels could be regulated to reduce memory dysfunction related to menopause, depression, and other conditions for which women are at greater risk.

## Introduction

The sex steroid hormone 17β-estradiol (E_2_) enhances hippocampus-dependent memory consolidation and promotes structural remodeling of dendritic spines in hippocampal area CA1. Although E_2_-driven increases in dendritic spine density and synaptic potentiation are well documented (Frick, 2015; Hojo et al., 2015; Hamson et al., 2016; Taxier et al., 2020), the molecular mechanisms that enable these structural and cognitive effects remain incompletely understood. Work to date has largely emphasized that E_2_ activates intracellular signaling cascades to promote new protein synthesis associated with synaptic remodeling. Our laboratory has shown that local protein synthesis in the dorsal hippocampus (DH) is necessary for E_2_ to increase CA1 spine density and enhance memory consolidation (Fortress et al., 2013; Tuscher et al., 2016), consistent with evidence that E_2_ activates rapid intracellular signaling cascades that promote translation at synapses (Akama and McEwen, 2003; Sarkar et al., 2010; Briz and Baudry, 2014; Sellers et al., 2015). However, long-term memory formation also depends on regulated protein degradation through the ubiquitin proteasome system (UPS), a critical pathway for synaptic remodeling that has been comparatively overlooked in the estrogenic memory literature (Beamish and Frick, 2022). This gap is significant because dynamic spine remodeling requires not only the addition of new proteins but also the timely removal of existing synaptic components. Thus, it is imperative to determine whether UPS-mediated protein degradation is necessary for E_2_ to enhance hippocampal memory consolidation and alter CA1 spine morphology.

The UPS is a primary pathway for intracellular protein degradation in mammalian cells (Glickman and Ciechanover, 2002). In this system, proteins are ubiquitinated with K48-linked polyubiquitin chains and targeted to the 26S proteasome for degradation. Although components of the UPS are expressed throughout neurons (Ádori et al., 2016), proteasome activity plays a particularly important role at synapses, where neuronal activity recruits proteasomes to spine heads and promotes degradation of synaptic scaffolding proteins to support structural and receptor-related remodeling (Ehlers, 2003; Bingol and Schuman, 2004, 2006; Hamilton et al., 2012). Accordingly, proteasomal protein degradation is a key regulator of long-term memory (Jarome and Helmstetter, 2013; Hegde, 2017), although most evidence for this role comes from studies in males. In male rodents, pharmacological inhibition of proteasome activity via intracranial infusion of drugs such as lactacystin and clasto-lactacystin-β-lactone (β-lac) into the hippocampus, amygdala, or prefrontal cortex disrupts phases of memory including consolidation, reconsolidation, extinction, and retrieval in fear, spatial, and object recognition tasks (Lopez-Salon et al., 2001; Artinian et al., 2008; Lee et al., 2008; Jarome et al., 2011; Rodriguez-Ortiz et al., 2011; Reis et al., 2013; Figueiredo et al., 2015; Cullen et al., 2017). Although a protein removal requirement may seem counterintuitive, degradation can facilitate memory by eliminating synaptic proteins that constrain remodeling, thereby permitting plasticity after learning.

UPS engagement during learning differs by sex and brain region, with males and females showing distinct patterns of proteasome activation, baseline ubiquitination, and dependence on UPS signaling following fear conditioning (Devulapalli et al., 2021; Martin et al., 2021; Farrell et al., 2023; Navabpour et al., 2024; Preveza et al., 2025; Turner et al., 2026). Sex differences in UPS recruitment have also been observed following object training (Beamish et al., 2022), providing important insight into how females engage protein degradation pathways during learning. Despite these advances, it remains unclear how sex steroid hormones like E_2_ regulate UPS activity in the hippocampus. Although limited, evidence indicates that E_2_ can modulate UPS signaling and influence degradation of synaptic proteins through estrogen receptor-dependent mechanisms in cultured hippocampal neurons (Zhang et al., 2011; Briz et al., 2015; Lai et al., 2019). Therefore, the present study tested whether protein degradation is required for E_2_ to enhance hippocampus-dependent memory consolidation and promote CA1 dendritic spine remodeling in female mice. Our data show that inhibiting proteasome activity blocks E_2_-mediated enhancements in memory and CA1 spine density and morphology in ovariectomized mice, providing the first evidence that protein degradation is necessary for estrogenic modulation of hippocampal memory and dendritic spine remodeling.

## Materials and Methods

### Subjects

Adult female C57BL6/NTac mice were purchased from Taconic Biosciences at 8–10 weeks of age. They were group housed (*n*  =  4–5/cage) upon arrival and were then single housed 24 h prior to surgery in shoebox cages in a vivarium (22–23°C) with a 12/12 h light/dark cycle. Food and water were provided *ad libitum*. Behavioral procedures were conducted from 10:00 to 17:00 h in a dimly lit quiet room. All procedures were approved by the University of Wisconsin–Milwaukee Institutional Animal Care and Use Committee following the National Institutes of Health Guide for the Care and Use of Animals.

### Surgeries

As is standard in the field, mice were bilaterally ovariectomized as described previously (Tuscher et al., 2016; Gross et al., 2022; 2021; Taxier et al., 2024) to pinpoint effects of exogenous E_2_ administration in the absence of confounds inherent to circulating ovarian hormone fluctuations. Immediately following ovariectomy, mice were implanted with custom cannulae (P1 Technologies) as per our previous work (Tuscher et al., 2018; Kim et al., 2019; Gross et al., 2022; 2021; Taxier et al., 2024) using a stereotaxic apparatus. Mice in Experiment 1 received bilateral cannulae targeting the DH (−1.7 mm A/P, ±1.5 mm M/L, −2.3 mm D/V), whereas mice in Experiments 2 and 3 received the same bilateral DH cannulae plus a third cannula targeting the dorsal third ventricle (ICV; −0.9 mm A/P, ±0.0 mm M/L, −2.3 mm D/V). Cannulae were secured in place with dental cement (Darby Dental), which also served to close the scalp incision.

Throughout surgery, mice were anesthetized with isoflurane (5% for induction, 3–2.5% for maintenance) in 100% oxygen. For analgesia, mice received a quarter of a 2 mg rimadyl tablet the day before, day of, and day after surgery. Mice also received a subcutaneous injection of rimadyl (10 ml/kg) at the start of surgery. All mice were monitored closely for 5 d postsurgery to ensure healthy recovery. Wound clips on ovariectomy sites were removed under anesthesia 1 week following surgery.

### Experiment 1: effects of β-lactone on memory consolidation

The role of proteasomal protein degradation in memory and E_2_-induced memory consolidation was tested using the proteasome inhibitor clasto-lactacystin-β-lactone (β-lac, Sigma-Aldrich), which inhibits the catalytic subunits of the 26S proteasome, thereby blocking proteasome activity. In studies of male rodents, dorsal hippocampal infusion of β-lac blocks the formation of spatial (Choi et al., 2010) and contextual fear memories (Cullen et al., 2017). Thus, we hypothesized that dorsal hippocampal proteasome activity would be necessary for object memory consolidation in ovariectomized female mice. To test this hypothesis, as well as identify a dose of β-lac that does not impair memory consolidation on its own to test interactions with E_2_ in subsequent experiments, ovariectomized mice in Experiment 1 were infused into the DH with vehicle or one of three doses of β-lac (8, 16, or 32 ng/µl) immediately after training in object recognition (OR) and object placement (OP) tasks. The 32 ng/µl dose was selected as a likely memory-impairing dose because infusion of this dose into the DH, amygdala, or prefrontal cortex of male rodents impaired spatial memory, as well as auditory and contextual fear memory consolidation (Choi et al., 2010; Jarome et al., 2011; 2016; Reis et al., 2013; Cullen et al., 2017). Post-training infusions were used here and in Experiment 2 to pinpoint effects of E_2_ and protein degradation to the memory consolidation phase of memory formation. Consolidation in these tasks occurs within 3 h after training (Frye et al., 2007; Fernandez et al., 2008) and immediate post-training DH infusions of E_2_ consistently enhance consolidation in both tasks in ovariectomized mice (Koss et al., 2018; Gross et al., 2021; 2022; Taxier et al., 2024).

### Experiment 1: drugs and infusions

Mice received a post-training bilateral DH infusion of vehicle (10% DMSO in sterile saline) or β-lac at one of three doses (4, 8, or 16 ng/hemisphere; equivalent to 8, 16, or 32 ng/µl in sterile saline). For intracranial infusions, mice were gently restrained, dummy cannulae were removed, and an infusion cannula (P1 Technologies; C313l; DH, 28 gauge; extending 0.8 mm beyond the 1.5 mm guide) was inserted. Infusion cannulae were connected to PE50 polyethylene tubing attached to a 10 µl Hamilton syringe, and drug delivery was controlled with a microinfusion pump (KDS Legato 180, KD Scientific). As per our previously described infusion protocol (Fortress et al., 2013; Gross et al., 2021; 2022), 0.5 µl of vehicle or β-lac was infused into each hemisphere of the DH at a rate of 0.5 µl/min. After each infusion, the infuser remained in place for 1 min to allow for diffusion of drugs into the tissue with minimal flow up the cannula track.

### Experiment 1: behavioral testing

OR and OP tasks were used as in our prior work to assess object recognition and spatial memory, respectively (Boulware et al., 2013; Tuscher et al., 2016; Gross et al., 2021). In both tasks, mice undergo a training session in which they freely explore two identical objects. After a delay, memory for the identity (OR) or location (OP) of the training objects is tested in a single session in which one object is replaced with a novel object (OR) or moved to a new location (OP). Because rodents are naturally inclined to explore novelty (Misslin, 1981), mice with intact memory should spend more time than chance exploring the novel or moved objects.

OR and OP were conducted in a white open field box (width, 60 cm; length, 60 cm; height, 47 cm). Prior to training, mice were handled for 30 s/day for 3 consecutive days to acclimate them to experimenters. On the second day of handling, a single Lego Duplo block was placed in the home cage to acclimate mice to objects. Following handling, mice underwent a single habituation session for 5 min/day for 2 consecutive days. During habituation, mice were allowed to move freely about the apparatus without objects present. Following habituation, mice underwent OR and OP training, during which mice were given up to 20 min to accumulate 30 s exploring two identical objects placed in the upper right- and left-hand corners of the box. An experimenter blind to treatment group scored object exploration manually in real time using the ANYmaze software (Stoelting). Object exploration was defined as the direction of the nose and/or front paws toward, or in contact with, the objects. Different objects were used for OR and OP, and all objects used were counterbalanced across mice to account for potential effects of object preference. If mice did not accumulate 30 s of exploration time during training, then they were then retrained 4–7 d later with different objects.

Immediately after successful accumulation of 30 s of exploration time with objects, mice received post-training bilateral DH infusion of vehicle or β-lac as described above. OR and OP testing occurred 24 and 4 h post-training, respectively. These delays between training and testing were chosen because our previous findings demonstrate that vehicle-infused mice remember the location and identity of training objects at these time points (Koss et al., 2018; Kim et al., 2019; Gross et al., 2021), thereby allowing us to determine if any doses of β-lac impaired memory consolidation relative to the vehicle group. Moreover, these delays permitted identification of at least one behaviorally subeffective dose of β-lac that could be used in Experiment 2 to test interactions with E_2_.

During OR and OP testing, mice were given 20 min to accumulate 30 s of exploration time with the objects. During OR testing, the least-explored training object was replaced by a novel object; mice that remember the identity of the training objects should spend more time than chance exploring the novel object. During OP testing, the least-explored training object was moved to the lower right- or left-hand corner of the apparatus; mice that remember the original location of the training objects should spend more time than chance exploring the moved object. Chance was designated at 15 s because this value represents equal exploration of both objects (Frick and Gresack, 2003). Time to accumulate 30 s of exploration was also recorded to assess whether group differences in motivation or activity levels contributed to treatment effects. Mice that failed to accumulate 30 s of exploration time during training were given up to three additional attempts to meet the criterion. Mice that did not meet the criterion were excluded from the study. All mice were tested in both tasks and were given at least 2 weeks between bouts of testing to ensure that any acute effects of previous infusions dissipated before the next treatment.

### Experiment 1: proteasome activity assays

To confirm that β-lac reduced proteasome activity in vivo and in vitro, we measured chymotrypsin-like 20S proteasome activity using a fluorescent substrate assay as described previously (Orsi et al., 2019; Beamish et al., 2022). For the in vivo assay, a subset of mice from Experiment 1 underwent a final object training session 1–2 weeks after completion of behavioral testing. Mice were trained with a novel set of objects and immediately received bilateral DH infusions of vehicle, 8 ng/µl β-lac, or 32 ng/µl β-lac (*n* = 5–9/group). The DH tissue was collected 1 h later. For the in vitro assay, adult female C57BL6/NTac mice (*n* = 6; 3/treatment) were ovariectomized, and the DH tissue was collected to assess the effectiveness of 25 µM β-lac at reducing proteasome activity, a concentration selected based on our previous work (Gross et al., 2021). Whole DH homogenates from both experiments were mixed with reaction buffer and incubated with or without 25 µM β-lac (in vitro assay only) together with the fluorogenic substrate Suc-Leu-Leu-Val-Tyr-AMC (Enzo) at 37°C to quantify chymotrypsin-like activity. Fluorescence (360 nm excitation/460 nm emission) was monitored every 30 min for 2 h using a microplate reader (Agilent Synergy H1). Fluorescence values were corrected using protein-free blanks, quantified against an AMC standard curve, and the scan exhibiting peak AMC fluorescence was used for analysis.

### Experiment 1: statistical analyses

All statistical analyses were performed using GraphPad Prism 10 (Version 10.4.0). To assess memory consolidation within each group, behavioral data were analyzed with one-sample *t* tests to determine if the time spent with the novel or moved object differed from chance (15 s). Between-group treatment effects on exploration time and time to accumulate 30 s of exploration were assessed using one-way ANOVA followed by Tukey's post hoc tests. To assess chymotrypsin-like proteasome activity levels following β-lac incubation, one-way ANOVA was used for the in vivo experiment, and an unpaired *t* test was used for the in vitro study. Statistical significance was determined at *p* ≤ 0.05 for all statistical tests, and trends were determined by *p* ≤ 0.10.

### Experiment 2: role of proteasome activity in E_2_-induced memory consolidation

This experiment tested the hypothesis that proteasome activity is necessary for the memory-enhancing effects of E_2_ by coinfusing the proteasome inhibitor β-lac in conjunction with E_2_. As in our previous studies involving multiple infusions (Kim et al., 2019; Gross et al., 2021; Taxier et al., 2024), bilaterally ovariectomized mice were implanted with bilateral cannulae into the DH for delivery of vehicle or β-lac and a third cannula targeting the dorsal third ventricle (ICV; −0.9 mm A/P, ±0.0 mm M/L, −2.3 mm D/V) for delivery of vehicle or E_2_. The resulting four groups of mice (*n* = 41; 9–11 per treatment) mice received DH and ICV infusions, respectively, of Veh + Veh, Veh + E_2_, β-lac + Veh, or β-lac + E_2_. To ensure that any effects of β-lac on estrogenic regulation of memory reflected a specific interaction with E_2_, rather than a nonspecific blockade of memory consolidation, we used the 8 ng/µl dose of β-lac identified in Experiment 1 that did not impair spatial or object recognition memory on its own. This strategy, which we routinely employ to demonstrate that behaviorally subeffective doses of inhibitors can block E_2_’s effects (Kim et al., 2019; Gross et al., 2021; Taxier et al., 2024), allowed us to directly test whether proteasome activity is required for E_2_-mediated memory enhancement.

### Experiment 2: drugs and infusions

Mice received a post-training bilateral DH infusion of vehicle (10% DMSO in sterile saline) or 4 ng/hemisphere β-lac at a rate of 0.5 μl/min for 1 min/hemisphere, as described above. Immediately following the DH infusion, mice received an ICV infusion of vehicle (2-hydroxypropyl-β-cyclodextrin in sterile saline; 10 µg/μl infused) or water-soluble β-cyclodextrin-encapsulated E_2_ (0.5 µl in sterile saline; 10 µg/μl infused). This triple infusion protocol was used to prevent tissue damage from multiple infusions into the DH in rapid succession (Fernandez et al., 2008; Zhao et al., 2010; Boulware et al., 2013; Fortress et al., 2013).

### Experiment 2: behavioral testing

The OR and OP tasks were completed as described above, except that these experiments were specifically designed to test the memory-enhancing effects of E_2_. As such, OR and OP testing occurred 48 and 24 h later, respectively. These timepoints were chosen because they are delays at which vehicle-infused mice do not display intact OR and OP memory consolidation (Koss et al., 2018; Kim et al., 2019; Gross et al., 2021), thereby allowing us to observe memory-enhancing effects of E_2_.

### Experiment 2: statistical analyses

To assess memory formation within each group, behavioral data were analyzed with one-sample *t* tests as in Experiment 1. For between-group comparisons, behavioral data were analyzed using a two-way ANOVA and Tukey post hoc tests with drug and hormone treatment as between-subject variables. Statistical significance was set at *p* ≤ 0.05 for all statistical tests, and trends were determined by *p* ≤ 0.10.

### Experiment 3: role of proteasome activity in E_2_-induced CA1 spine density and morphology

Experiment 3 tested the hypothesis that proteasome activity is necessary for E_2_ to increase CA1 dendritic spine density and morphology in the DH. Mice (*n* = 25; 6–7 per treatment) were bilaterally ovariectomized and implanted with triple guide cannulae targeting the DH and ICV, as described for Experiment 2. Following surgical recovery, mice were handled to acclimate them to experimenters and then received a single DH and ICV infusion, respectively, of Veh + Veh, Veh + E_2_, β-lac + Veh, or β-lac + E_2_, following the same procedures as in Experiment 2.

### Experiment 3: tissue preparation and Golgi staining

Whole brains were collected 2 h after infusion via cervical dislocation and decapitation for analysis of CA1 spine density. This time point was chosen based on our previous findings that ICV infusion of E_2_ increases CA1 dendritic spine density 2 h later (Tuscher et al., 2016). Golgi staining was performed using the Rapid Golgi Stain Kit (FD NeuroTechnologies) as described previously (Tuscher et al., 2016; Kim et al., 2019; Taxier et al., 2022). Immediately after collection, brains were immersed in the kit’s impregnation solution at room temperature in the dark for 2 weeks. Following impregnation, the tissue was flash frozen in isopentane on dry ice and stored at −80°C. Brains were then sectioned into 100-µm-thick slices using a Leica CM3050 cryostat at −30°C and mounted onto gelatin-coated microscope slides. Sections were stained according to the manufacturer's instructions, coverslipped with Permount solution and then allowed to dry in the dark for 2 weeks prior to imaging. Slides were coded so that the experimenter was blind to animal number and treatment. When not being analyzed, Golgi-stained sections were stored in slide boxes to protect them from light exposure.

### Experiment 3: neuron selection and imaging

Representative pyramidal neurons with clearly visible apical and basal dendrites were selected from the CA1 region of the DH, based on previously established criteria (Frankfurt et al., 2011; Tuscher et al., 2016; Kim et al., 2019; Taxier et al., 2022). All tracings and images were acquired using an Olympus BX51WI microscope and Neurolucida software (MBF Bioscience, version 2024.1.3). At 2× magnification, area CA1 was outlined for each section of the tissue on the slide. At 10×, all cell bodies appearing to have countable dendritic segments were traced. Neurons were included for *z*-stack imaging if they met the following criteria: (1) centrally located within the tissue depth, (2) free of staining debris or overlapping neurons, and (3) completely impregnated by Golgi stain. *Z*-stack images of selected neurons were then captured at 60× oil immersion at 0.5 µm intervals along the *z*-axis, beginning when the cell first appeared and ending when it disappeared. Due to variability in Golgi staining, not all neurons exhibited complete staining of both basal and apical dendrites, so neurons with basal and apical dendrites were imaged separately. Six neurons per brain per dendrite type were analyzed, yielding a total of 150 neurons for basal dendrite analysis and 150 neurons for apical dendrite analysis.

### Experiment 3: neuron reconstruction

Neurolucida 360 (MBF Bioscience, version 2024.2.2) was used to generate 3D reconstructions of selected neurons, including soma, dendritic arborization, and dendritic spines. Individual image stacks were imported and stitched into composite stacks for analysis. To minimize out-of-focus signal in the bright-field images, composite stacks were processed using the software's recommended Golgi filter settings at 100–150% sensitivity.

Structures were traced in the 3D window of Neurolucida 360 as described previously (Rodriguez et al., 2008; Dickstein et al., 2016). The soma was modeled by selecting the cell body with detector sensitivity set to 50–75%. Basal and apical processes were traced using the semiautomatic, user-guided feature, which detects process thickness and path between user-defined points. The directional kernels tracing method was applied, in which four orientation-specific kernels (top, bottom, left, right) were matched to the image data. At each point, the algorithm integrated the best kernel orientations to estimate the next tracing point, continuing until stopping criteria were met by the user. This approach enhanced detection of dendrites by highlighting edges aligned with each orientation while allowing user guidance to direct the tracings. As such, the basal and apical dendrites were manually traced, with dendrite thickness recorded and branching order tracked by placing nodes at branch points within the dendritic tree.

### Experiment 3: dendritic segment selection

Following reconstruction of the entire neuron, two sets of markers spaced 20 µm apart were placed on each cell. One set defined a 20 µm segment on a tertiary apical dendrite and the second defined a different 20 µm segment on another tertiary apical branch of the same neuron. The same procedure was repeated for secondary basal dendrites, resulting in two predefined basal segments per neuron.

Tertiary apical and secondary basal dendrites were defined according to the shaft-ordering scheme, which identifies one primary dendrite as the dominant path and classifies all other branches as derivative to it, consistent with our previous work (Tuscher et al., 2016; Kim et al., 2019; Taxier et al., 2022). These branch orders were selected to provide standardized sampling across neurons while minimizing variability in spine measurements. Segments were included only when dendritic staining was intact, traces could be cleanly reconstructed in 3D, and sampled segments originated from distinct dendritic trees. As such, a total of 12 dendritic segments per brain were analyzed or each dendrite type.

### Experiment 3: arbor-wide spine detection

Following placement of segment markers, dendritic spines were detected across the entire reconstructed neuron using Neurolucida 360’s automated spine detection algorithm. Identical detection parameters were applied to every neuron using the program’s default settings (outer range, 2.5 µm; minimum height, 0.3 µm; detector sensitivity, 100%; minimum count, 10 voxels).

### Experiment 3: segment-based spine verification and classification

Following automated spine detection across the entire reconstructed arbor, the reconstruction file was duplicated so that one version could be retained for arbor-wide analyses. In the duplicate file, every spine located within the predefined 20 µm tertiary apical and secondary basal dendritic segments was manually inspected to verify accurate reconstruction and identify errors resulting from axial smear or incorrect segmentation, as described previously (Walker et al., 2022; 2023; 2024). Each spine was evaluated by toggling tracing mode on and off to confirm accurate overlay with the underlying image in the XY, XZ, and YZ planes. When necessary, individual spines were merged, split, deleted, or redetected until each segment was accurately reconstructed.

Following manual verification, spines within the predefined dendritic segments were automatically classified as stubby, mushroom, or thin using the program’s default classification parameters developed for mouse hippocampal neurons (Dickstein et al., 2016; Rodriquez et al., 2008; head/neck ratio, 1.1; length/head ratio, 2.5; mushroom head size, 0.35 μm).

This workflow follows the Neurolucida 360 methodology described by Walker et al. (2022; 2023; 2024), in which representative dendritic segments rather than the entire dendritic arbors were manually reconstructed and verified prior to quantitative and morphometric analyses. Consequently, spine subtype classification and three-dimensional morphometric measurements were restricted to manually verified spines within the predefined dendritic segments.

### Experiment 3: dendritic morphology and spine analyses

Dendritic morphology and spine measurements were quantified using Neurolucida Explorer (MBF Bioscience, version 2025.2.1) using two complementary analytical approaches: an arbor-wide analysis to quantify global dendritic architecture and spine distribution and a segment-based analysis to obtain manually verified spine subtype and three-dimensional morphometric measurements.

The arbor-wide analysis quantified spine density across all reconstructed basal and apical dendrites, providing a global measure of spine density throughout the reconstructed arbor. Arbor-wide analyses also included Sholl analyses (Sholl, 1953) to assess radial spine distribution and dendritic complexity. In Neurolucida Explorer, concentric spheres centered on the soma were generated at successive 10 µm intervals, and the numbers of dendritic spines, intersections, nodes, surface area, and diameter at each radius were quantified. To ensure equivalent sampling across neurons, Sholl analyses were truncated to the maximum radial distance common to all reconstructed neurons, such that all cells contributed the same number of radial bins. Thus, the Sholl analyses provided an arbor-wide assessment of how spine and dendritic measures varied as a function of radial distance from the soma.

The segment-based analysis quantified spine density, spine subtype density, and detailed three-dimensional spine morphology within the predefined dendritic segments. In contrast to the Sholl analyses, these measurements were performed on standardized shaft-defined branch-order categories and therefore provide information complementary to, rather than directly overlapping with, the radial measurements obtained from Sholl analyses. Importantly, these branch-order categories do not necessarily correspond to specific proximal or distal dendritic compartments. It is important to note that the axial and lateral resolution of bright-field microscopy is not as fine as super-resolution methods such as electron microscopy, and in vivo Golgi staining underestimates spine density and overestimates spine volume and spine head diameter relative to iontophoretic microinjection of Lucifer yellow (Walker et al., 2022). As such, our Golgi-based estimates are best understood as a “geometric extrapolation” of spine morphology. However, because all tissue in the present study was processed, imaged, reconstructed, and analyzed using identical acquisition parameters and analysis settings, systematic bias inherent to bright-field Golgi imaging would be expected to affect all experimental groups equally. Consequently, our conclusions are based on relative differences between treatment groups rather than absolute morphometric values.

### Experiment 3: statistical analyses

For the arbor-wide analysis, spine density was calculated by dividing the total number of spines across all reconstructed dendritic branches by the total dendritic length of the reconstructed arbor and multiplying by 10 to express density as spines per 10 µm. Arbor-wide spine density, area under the curve (AUC), and cell body metrics were analyzed using two-way ANOVAs with hormone and drug as between-subject factors, followed by Tukey's post hoc tests where appropriate.

Sholl analyses were performed on reconstructed apical and basal dendritic arbors using 10 µm radial intervals from the soma. Spine number and dendritic intersections are presented as primary outcomes in the main text, whereas additional Sholl-derived measures (dendritic length, surface area, volume, and node number) are reported in Supplemental Material. Consistent with the procedures described above, all Sholl analyses were restricted to the radial range common to all reconstructed neurons, regardless of treatment. Sholl data were analyzed using three-way mixed-effects models with distance as the repeated-measures factor and hormone and drug as between-subject factors, followed by Tukey's post hoc tests, as described previously (Wilson et al., 2017).

For the segment-based analysis, spine density was calculated by dividing the total number of spines counted across 12 sampled tertiary segments per animal by the total length of those segments and multiplying by 10 to express density as spines per 10 µm. Segment spine density and dendritic segment morphological measures were analyzed using two-way ANOVAs with hormone and drug as between-subject factors, whereas spine-type analyses were analyzed using three-way ANOVAs with spine type, hormone, and drug as factors, followed by Tukey's post hoc tests where appropriate. Statistical significance was set at *p* ≤ 0.05 for all analyses, and trends were defined as *p* ≤ 0.10.

## Results

### β-Lactone dose-dependently impaired hippocampal memory consolidation

Because proteasomal protein degradation is an enzymatically driven process, we opted for a pharmacological approach to demonstrate the importance of this process for estrogenic regulation of memory and hippocampal spine morphology. The proteasome inhibitor β-lac was the ideal compound for this purpose because it blocks catalytic activity of the 26S proteasome. We first tested the effects of DH β-lac infusion on memory consolidation in the OR and OP tasks (Fig. 1*A*). Immediately after training in OR (Fig. 1*B*) and OP (Fig. 1*C*), OVX mice received DH infusion of vehicle or one of three doses of β-lac (8, 16, or 32 ng/µl), and memory was tested 24 and 4 h later, respectively. During OR testing, mice receiving DH infusion of vehicle (*t*_(9)_ = 2.317; *p* = 0.0457) or 8 ng/µl β-lac (*t*_(10)_ = 3.237; *p* = 0.0089) immediately after training spent significantly more time than chance (15 s) with the novel (Fig. 1*D*) object, whereas mice treated with 32 ng/µl β-lac did not. Mice treated with 16 ng/µl β-lac (*t*_(7)_ = 4.176; *p* = 0.0042) spent significantly less time than chance with the moved object, suggesting a possible aversion to the novel object. Importantly, these data indicate that 16 and 32 ng/µl β-lac impaired OR memory consolidation whereas 8 ng/µl did not. Accordingly, the main effect of treatment was significant (*F*_(3,32)_ = 6.068; *p* = 0.0022), with post hoc comparisons indicating that the 16 ng/µl β-lac group spent less time with the novel object than the vehicle (*p* = 0.0089) or 8 ng/µl β-lac (*p* = 0.0035) groups. Treatment did not affect the time to accumulate 30 s exploration with objects during testing (Fig. S1*A*), suggesting no effect of motivation or activity to explore objects across treatments.

**Figure 1.**
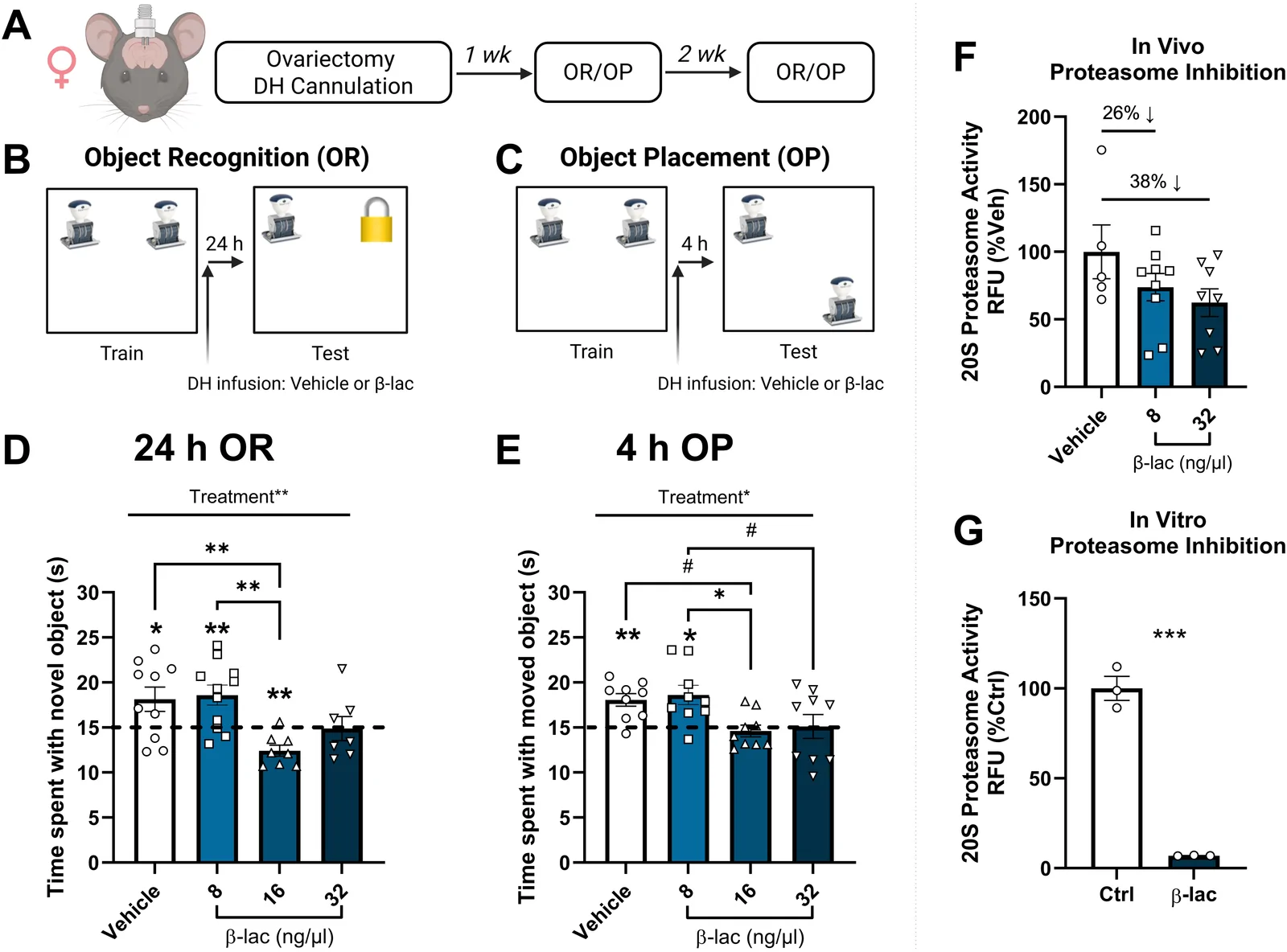
β-Lac dose-dependently blocked spatial and object memory consolidation in OVX mice. ***A–C***, Study design, *n* = 7–11/group. One week after ovariectomy and DH cannulation surgery, mice were trained and tested in OR or OP, followed 2 weeks later by training and testing in the other task (order of testing was counterbalanced within each group). Immediately after training, mice were infused with vehicle or one of three doses of β-lac (8, 16, 32 ng/µl) and then were tested 24 h later in OR and 4 h later in OP, delays at which vehicle-infused mice show intact memory consolidation by spending more time than chance with the novel and moved objects. ***D***, In the OR task, the vehicle (*p < 0.05) and 8 ng/µl (**p < 0.01) groups spent more time than chance (15 s, dashed line) with the novel object when tested 24 h after training, whereas the 16 ng/µl group (**p < 0.01) spent less time with the novel object. The 32 ng/µl group did not differ from chance. Accordingly, the main effect of treatment was significant, and post hoc tests showed that vehicle and 8 ng/µl groups spent more time exploring the novel object than the 16 ng/µl group (**p < 0.01). ***E***, In the OP task, the vehicle (**p < 0.01) and 8 ng/µl (*p < 0.05) groups spent more time than chance with moved object 4 h after training, whereas neither the 16 nor 32 ng/µl groups differed from chance. Again, the main effect of treatment was significant, and the 16 ng/µl β-lac group spent less time with the moved object than mice receiving 8 ng/µl β-lac (*p < 0.05). Vehicle and 8 ng/µl β-lac groups tended to spend more time with the moved object than 16 and 32 ng/µl groups, respectively (^#^*p* < 0.10). ***F***, To assess whether the doses used in the behavioral experiments inhibited proteasome activity in vivo, mice received immediate post-training DH infusions of vehicle, 8 ng/µl, or 32 ng/µl β-lac, and the DH tissue was collected 1 h later for measurement of 20S chymotrypsin-like activity (*n* = 5–9/group). Both β-lac doses produced numerical decreases in activity relative to vehicle (8 ng/µl, ∼26%; 32 ng/µl, ∼38%), although these differences did not reach statistical significance. ***G***, 25 µM β-lac (∼5.3 ng/µl) significantly reduced chymotrypsin-like activity in whole-cell DH homogenate collected from OVX female mice (***p < 0.001). Error bars indicate mean ± standard error of the mean (SEM).

Results for spatial memory mirrored those of object recognition memory. Mice receiving DH infusion of vehicle (*t*_(8)_ = 4.380; *p* = 0.0023) or 8 ng/µl β-lac (*t*_(8)_ = 3.339; *p* = 0.0102) immediately after training spent significantly more time than chance (15 s) with the moved object in the OP task (Fig. 1*E*), indicating intact spatial memory consolidation in these groups. In contrast, mice receiving 16 or 32 ng/µl β-lac did not spend more time than chance with the moved object, suggesting that these doses impaired spatial memory consolidation. These conclusions were supported by one-way ANOVA and post hoc tests, which revealed a main effect of treatment (*F*_(3,32)_ = 4.278; *p* = 0.0120), driven largely by the 8 ng/µl β-lac group spending more time with the moved object than the 16 ng/µl β-lac group (*p* = 0.0339). Vehicle-treated mice also tended to spend more time with the moved object than those treated with 16 ng/µl β-lac (*p* = 0.0815). Similarly, the 8 ng/µl β-lac group tended to spend more time with the moved object than the 32 ng/µl β-lac group (*p* = 0.0749). As with OP, treatment did not affect time to accumulate 30 s of object exploration (Fig. S1*B*).

We next examined whether the behaviorally ineffective 8 ng/µl and memory-impairing 32 ng/µl doses of β-lac used in our behavioral experiments reduced in vivo proteasome activity. Compared with vehicle-treated controls, mice receiving 8 ng/µl β-lac exhibited a 26% reduction in chymotrypsin-like 20S proteasome activity, whereas mice receiving 32 ng/µl β-lac exhibited a 38% reduction (Fig. 1*F*), although these reductions did not reach statistical significance (Data S1), perhaps due to issues related to diffusion and dilution in the DH tissue that could have affected measurement at the 1 h time point. Thus, we next incubated DH whole-cell lysates in vitro with 25 µM β-lac (∼5.3 ng/µl, a lower concentration than the 8 ng/µl dose used in vivo), which reduced chymotrypsin-like 20S proteasome activity by ∼93% relative to vehicle (Fig. 1*G*; *t*_(4)_ = 14.0; *p* = 0.0002), suggesting that β-lac does inhibit proteasome activity in DH tissue.

Together, the results of this dose–response study reveal that β-lac dose-dependently impaired object recognition and spatial memory consolidation. The 16 and 32 ng/µl doses blocked object recognition and spatial memory consolidation, suggesting that DH proteasomal protein degradation is necessary for object recognition and spatial memory formation in OVX mice. In contrast, the 8 ng/µl dose did not impair memory consolidation in either task, which made it the ideal dose to combine with E_2_ in our next experiments to examine the role of proteasomal protein degradation to estrogenic regulation of memory consolidation and CA1 dendritic morphology. The use of a behaviorally subeffective dose of β-lac ensured that any effects on memory seen in mice treated with E_2_ and β-lac could be attributed to their interaction rather than an inhibitory effect of β-lac alone on memory. We use this approach routinely to show that behaviorally subeffective doses of inhibitors and antagonists block the effects of E_2_ (Tuscher et al., 2016; Kim et al., 2019; Gross et al., 2021).

### E_2_ requires proteasomal protein degradation to enhance hippocampal memory consolidation

We next tested the extent to which proteasomal protein degradation is necessary for E_2_ to enhance memory consolidation in the OR and OP tasks. OVX mice were implanted with bilateral cannulae into the DH to deliver vehicle or β-lac (8 ng/µl at 4 ng/hemisphere) and an ICV cannula to deliver vehicle (Veh) or 10 µg E_2_ (Fig. 2*A*) immediately after training in OR (Fig. 2*B*) or OP (Fig. 2*C*). Thus, mice received DH and ICV infusions, respectively, of Veh + Veh, Veh + E_2_, β-lac + Veh, or β-lac + E_2_. Memory was tested 48 h (OR) or 24 h (OP) later, delays at which vehicle-infused mice do not display intact OR and OP memory consolidation, thereby allowing us to observe memory-enhancing effects of E_2_ (Tuscher et al., 2016; Kim et al., 2019; Gross et al., 2021).

**Figure 2.**
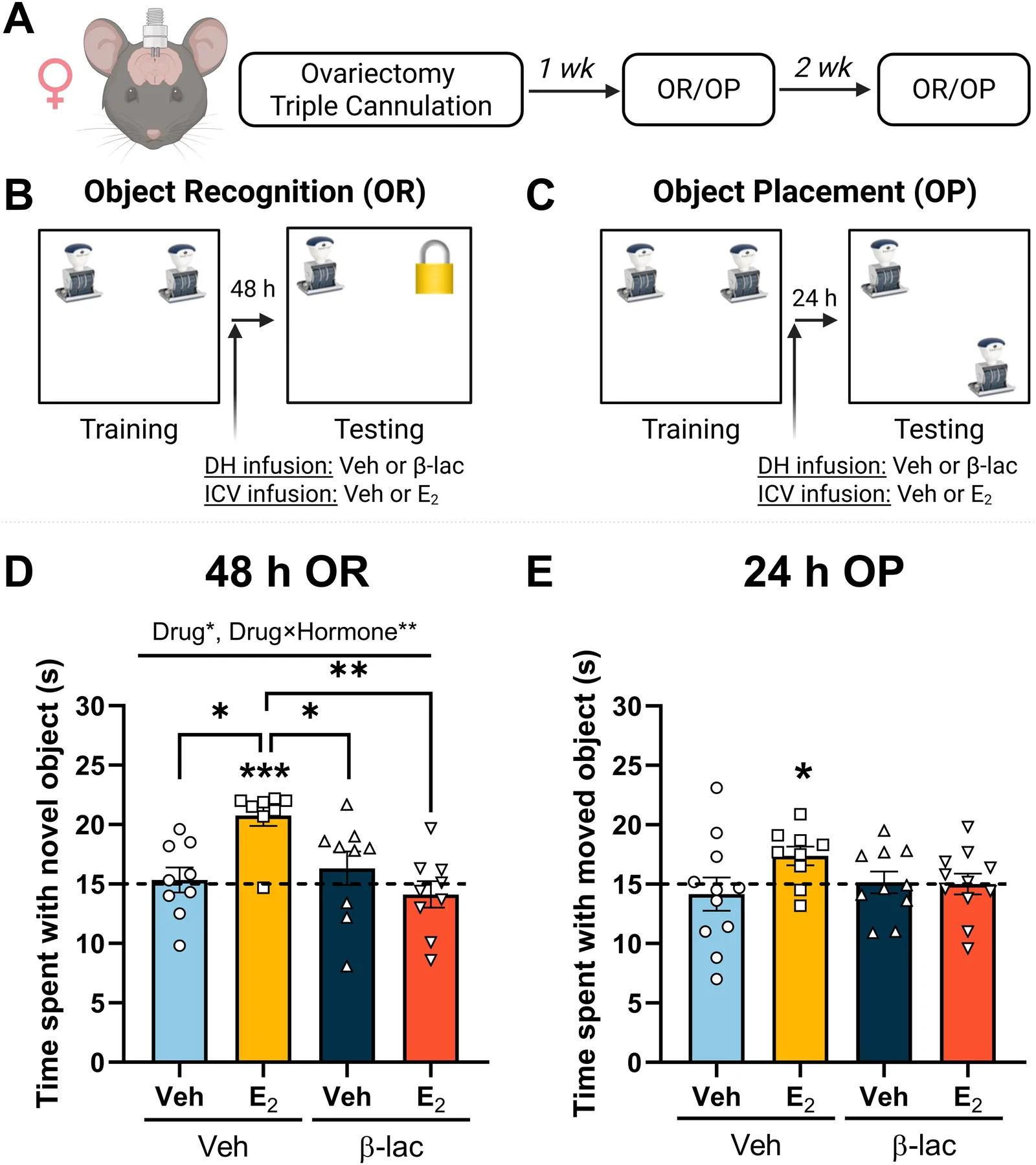
β-Lac blocked the object recognition memory-enhancing effects of E_2_ and attenuated the spatial memory-enhancing effects of E_2_ in OVX mice. ***A–C***, Study design, *n* = 9–11/group. One week before behavioral testing, mice were ovariectomized and implanted with a triple cannula mount containing bilateral DH cannulae and a dorsal third ventricle (ICV) cannula. Immediately after training, mice received DH infusion of vehicle or β-lac (4 ng/hemisphere) and ICV infusion of vehicle or E_2_ (10 µg) and then were tested 48 h later in OR and 24 h later in OP, delays at which vehicle-infused mice do not exhibit intact memory. ***D***, In OR, mice treated with Veh + E_2_ spent more time than chance with the novel object (***p < 0.001), whereas all other groups did not. Treatment significantly affected OR, driven by differences between Veh + E_2_-treated mice and all other groups (*p < 0.05; **p < 0.01). ***E***, In OP, mice treated with Veh + E_2_ also spent more time than chance with the moved object (*p < 0.05), whereas all other groups did not. Error bars indicate mean ± SEM.

During OR testing, the Veh + E_2_ group spent significantly more time than chance with the novel object during testing (Fig. 2*D*; *t*_(7)_ = 6.52; *p* = 0.0003), whereas all other groups did not, suggesting impaired object recognition memory among all but the Veh + E_2_-treated group. There was a significant main effect of DH drug treatment (vehicle or β-lac; *F*_(1,31)_ = 6.13; *p* = 0.0190), no main effect of ICV hormone treatment (vehicle or E_2_; *F*_(1,31)_ = 2.01; *p* = 0.1664), and a significant interaction between drug and hormone treatment (*F*_(1,31)_ = 11.16; *p* = 0.0022). This interaction was driven by the Veh + E_2_ group, which spent significantly more time with the novel object than the Veh + Veh (*p* = 0.0119), β-lac + E_2_ (*p* = 0.0017), and β-lac + Veh (*p* = 0.05) groups. Neither drug nor hormone affected the time to accumulate 30 s of exploration time at test (Fig. S1*C*).

During OP testing, the Veh + E_2_ group spent significantly more time than chance (15 s) with the moved object during testing (Fig. 2*E*; *t*_(8)_ = 3.02; *p* = 0.0165), whereas all other groups did not, suggesting that only the Veh + E_2_ group demonstrated evidence of spatial memory. However, unlike OR, there were no main effects of drug or hormone or their interaction, indicating that treatment effects on OP memory were less robust. Time required to accumulate 30 s of object exploration during testing did not differ among groups, suggesting that activity or motivation to explore objects was not affected by treatment (Fig. S1*D*).

Collectively, these findings replicate previous reports that DH E_2_ infusion can enhance OR and OP memory consolidation in OVX mice (Koss et al., 2018; Tuscher et al., 2018; Gross et al., 2022; 2021; Taxier et al., 2024). In contrast, the β-lac + E_2_ group showed no evidence of learning in either task and were significantly impaired relative to the Veh + E_2_ group in OR. Together, these findings suggest that proteasomal protein degradation contributes to E_2_-induced enhancement of object recognition memory and may similarly support E_2_-associated improvements in spatial memory in OVX mice.

### E_2_ broadly enhances spine density across the apical arbor and produces more modest parallel effects across the basal arbor

#### CA1 apical dendritic arbor

To determine whether E_2_ alters apical spine density independently of gross neuronal morphology, the soma of each neuron used for apical spine analysis was modeled in 3D. Average soma length (Fig. S2*A*), volume (Fig. S2*B*), and surface area (Fig. S2*C*) did not differ by drug, hormone, or their interaction (Data S1).

Previous studies examining E_2_-induced changes in dendritic spine density (Tuscher et al., 2016; Kim et al., 2018; Taxier et al., 2020) have been limited to manually defined dendritic segments because quantifying spines across the entire dendritic arbor is technically challenging. Using automated spine detection following full neuronal reconstructions, we were able to quantify E_2_-induced changes in spine density across the CA1 dendritic arbor (Fig. S3*A*–*D*; Movie 1). Quantification of arbor-wide apical spine density (spines/10 µm) revealed a significant main effect of drug (Fig. 3*B*; *F*_(1,20)_ = 5.024; *p* = 0.0365), with no main effect of hormone or drug × hormone interaction (Data S1), indicating that β-lac reduced overall apical spine density regardless of hormone condition. Post hoc analysis revealed that arbor-wide apical spine density was ∼14.4% higher in the Veh + E_2_ group than in the β-lac + Veh group (*p* = 0.0434) and tended to be ∼14.1% higher than in the β-lac + E_2_ group (*p* = 0.0605).

**Figure 3.**
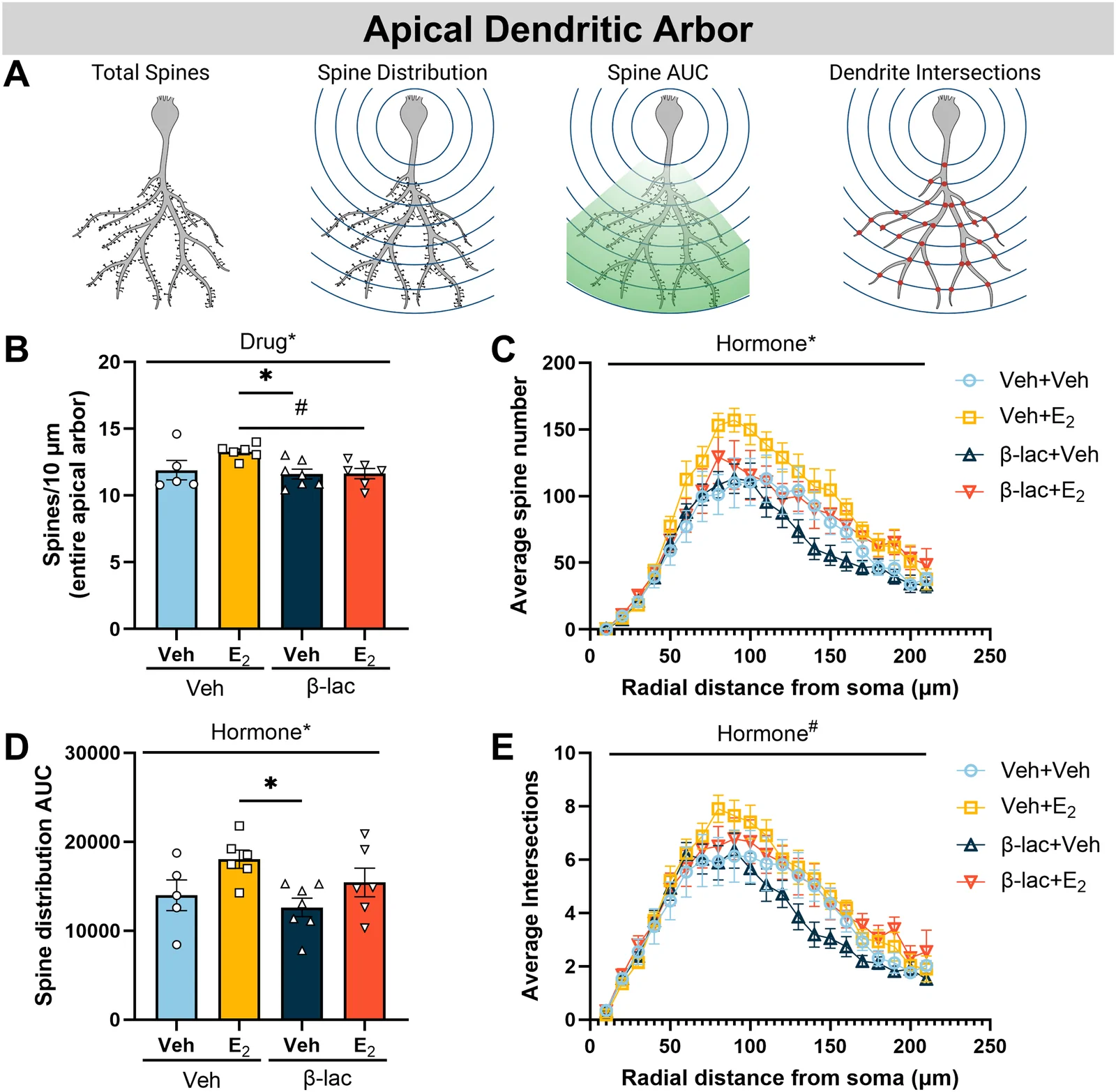
E_2_ enhanced overall apical spine density and increased spine number across the apical dendritic arbor. ***A***, Schematic illustrating experimental measures used to quantify apical dendritic spine organization, including total apical spine density, radial spine distribution, spine distribution AUC, and apical dendritic intersections (*n* = 25 brains; 5–7 per treatment group; 6 neurons per brain; total *n* = 150 apical dendrite-bearing neurons). ***B***, Veh + E_2_ treatment increased apical spine density relative to β-lac + Veh (*p < 0.05), whereas β-lac + E_2_-treated mice tended to exhibit reduced spine density compared with Veh + E_2_-treated mice (^#^*p* < 0.10), contributing to a main effect of drug treatment. ***C***, Radial spine distribution analysis revealed a main effect of hormone, indicating that E_2_ increased average spine number broadly across the apical arbor rather than at discrete distances from the soma. ***D***, Consistent with panel ***C***, AUC analysis demonstrated greater overall spine number across the apical arbor in E_2_-treated mice, including an increase in Veh + E_2_-treated mice relative to β-lac + Veh-treated mice that contributed to a main effect of hormone (*p < 0.05). ***E***, E_2_ treatment tended to increase the average number of dendritic intersections across the apical arbor (^#^*p* < 0.10). Error bars represent mean ± SEM.

**Movie 1.** Representative apical dendritic arbor reconstruction. Representative CA1 apical dendritic arbor illustrating the Neurolucida 360 reconstruction workflow shown in Figure S3*A*–*D*. The video demonstrates the reconstructed neuron with dendritic and spine overlays toggled on and off to visualize user-guided dendritic tracing and automated spine detection across the reconstructed arbor. [View online]

Because arbor-wide spine density does not provide information about where along the dendritic tree spines are altered, we next conducted a Sholl analysis to assess whether E_2_ changed the spatial distribution of apical spines as a function of distance from the soma (Fig. 3*C*). This analysis revealed that E_2_ increased apical spine number broadly across radial distances, rather than producing radius-specific changes along the apical dendritic tree. As expected, spine count varied significantly across radii (*F*_(3.512,70.24)_ = 86.90; *p* < 0.0001), indicating that apical spine number naturally changed as a function of distance from the soma. There was also an effect of hormone (*F*_(1,20)_ = 6.592; *p* = 0.0184), whereas the main effect of drug and all remaining interactions were not significant (Data S1), consistent with a broad effect of E_2_ across the apical arbor. To provide an integrated measure of spine distribution across radial distances, we calculated the area under the Sholl curve (AUC) for each neuron (Fig. 3*D*). AUC differed significantly by hormone (*F*_(1,20)_ = 6.493; *p* = 0.0192), but not by drug or drug × hormone interaction (Data S1). Post hoc analysis indicated that this effect was driven by differences between the Veh + E_2_ and β-lac + Veh groups, with the Veh + E_2_ group exhibiting a ∼43.0% greater AUC (*p* = 0.0348). Together, the Sholl and AUC analyses indicate that E_2_ increased apical spine number broadly across the analyzed radial range, resulting in an overall upward shift in radial spine distribution rather than selective remodeling at specific radial distances. This increase, however, was not dependent on protein degradation, given the lack of drug effects in both analyses.

To assess treatment effects on dendritic branching complexity, apical intersections were quantified across the reconstructed apical arbor (Fig. 3*E*). As expected, dendritic intersections varied significantly across apical radii (*F*_(3.263, 68.52)_ = 86.01; *p* < 0.0001). There was a trend for a main effect of hormone (*F*_(1,21)_ = 3.269; *p* = 0.0849), but the main effect of drug and all remaining interactions were not significant (Data S1). Additional apical Sholl-derived morphometric measures showed no hormone effect on dendritic volume (Fig. S4*A*; µm^3^), but trending effects on dendritic surface area (Fig. S4*B*; *F*_(1,21)_ = 3.006; *p* = 0.0976; µm^2^) and length (Fig. S4*C*; *F*_(1,21)_ = 3.133; *p* = 0.0913; µm), with no effect on the number of nodes (Fig. S4*D*). No other main effects and interactions were significant (Data S1). These findings indicate that E_2_-related differences in apical spine number were not driven by major changes in dendritic branching complexity, although E_2_ may have modestly influenced aspects of apical arborization.

#### CA1 basal dendritic arbor

Compared with the apical arbor, treatment effects in the basal arbor were generally smaller and less robust, although several measures exhibited qualitatively similar trends. Basal dendritic arbors were fully reconstructed and analyzed using the same procedures described for apical dendrites (Fig. 4*A*; Fig. S3*E*–*H*; Movie 2). Similar to apical neurons, average soma length (Fig. S2*D*), volume (Fig. S2*E*), and surface area (Fig. S2*F*) did not differ by drug, hormone, or their interaction in neurons used for basal spine analyses (Data S1).

**Figure 4.**
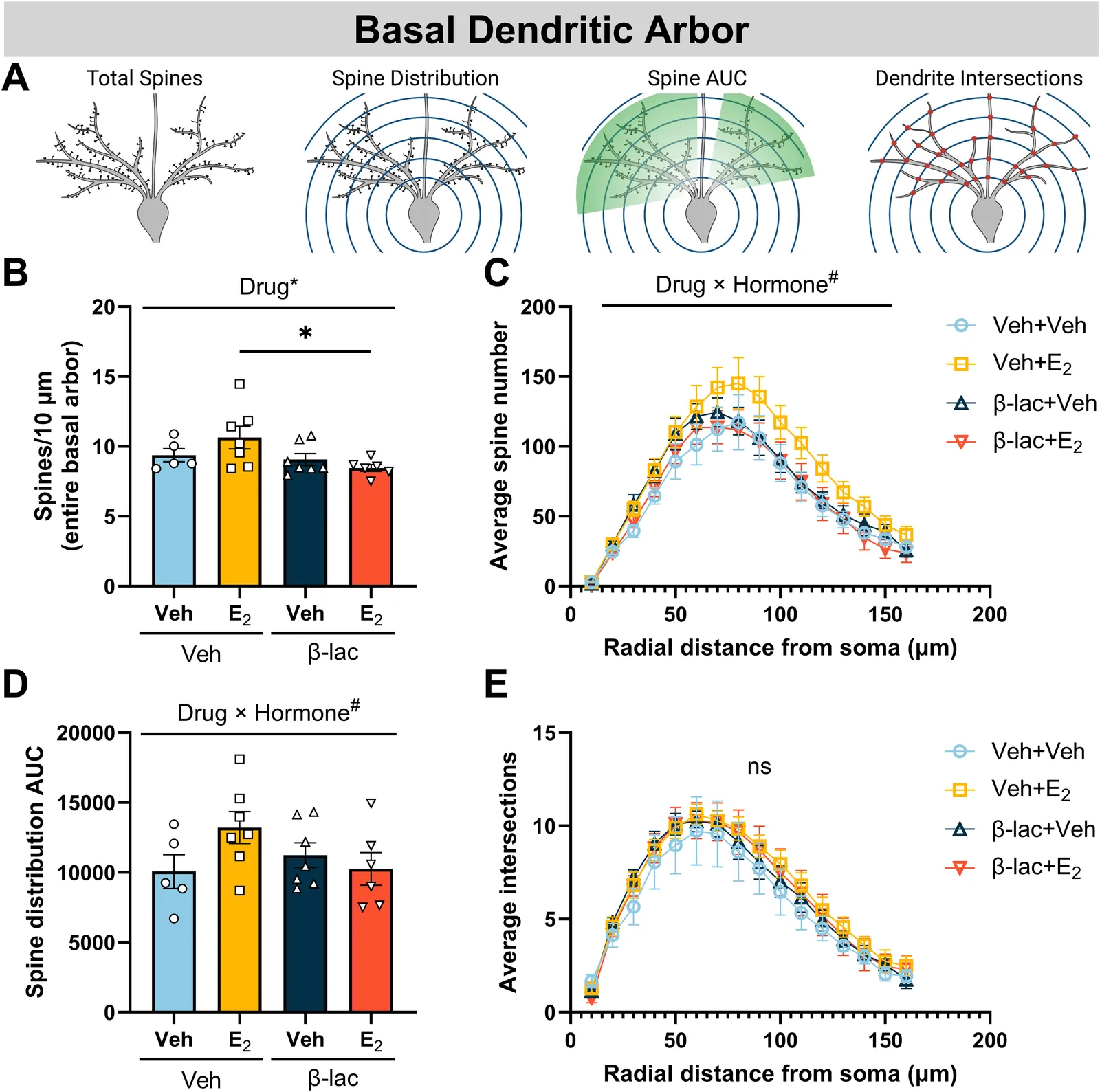
E_2_ modestly enhanced overall basal spine density and induced smaller, more localized increases in spine number across the basal dendritic arbor. ***A***, Schematic illustrating experimental measures used to quantify basal dendritic spine organization, including total basal spine density, radial spine distribution, spine distribution AUC, and basal dendritic intersections (*n* = 25 brains; 5–7 per treatment group; 6 neurons per brain; total *n* = 150 basal dendrite-bearing neurons). ***B***, β-lac treatment reduced basal spine density, driven in part by a significant reduction in basal spine density in the β-lac + E_2_ group relative to the Veh + E_2_ group (*p < 0.05). ***C***, Radial spine distribution analysis revealed a trend toward a drug × hormone interaction, such that E_2_ increased average spine number across portions of the basal arbor in vehicle-treated mice, an effect that tended to be reduced by proteasome inhibition (^#^*p* < 0.10). ***D***, Consistent with panel ***C***, AUC analysis of basal spine distribution showed a trend toward increased overall spine number across the basal arbor in Veh + E_2_-treated mice (^#^*p* < 0.10), although this effect did not reach statistical significance. ***E***, The average number of basal dendritic intersections did not differ by hormone or drug treatment (ns). Error bars indicate mean ± SEM.

**Movie 2.** Representative basal dendritic arbor reconstruction. Representative CA1 basal dendritic arbor illustrating the Neurolucida 360 reconstruction workflow shown in Figure S3*E*–*H*. [View online]

Arbor-wide basal spine density differed significantly by drug (Fig. 4*B*; *F*_(1,21)_ = 4.851; *p* = 0.0389), but not by hormone or drug × hormone interaction (Data S1), indicating that β-lac reduced basal spine density regardless of hormone condition. Post hoc analysis revealed that the Veh + E_2_ group exhibited ∼20.5% greater spine density than the β-lac + E_2_ (*p* = 0.0486) group.

As observed across the apical arbor, basal spine number varied significantly across radii (Fig. 4*C*; *F*_(2.272,47.71)_ = 110.7; *p* < 0.0001). There was a trend toward a drug × hormone interaction (*F*_(1,21)_ = 3.586; *p* = 0.0721), whereas the main effects of drug and hormone and all remaining interactions were not significant (Data S1). Similarly, AUC did not differ significantly by hormone or drug (Fig. 4*D*), although a trend toward a drug × hormone interaction was observed (*F*_(1,21)_ = 3.501; *p* = 0.0753). Together, these findings suggest a modest attenuation of E_2_-associated increases in basal spine distribution by β-lac.

Dendritic intersections varied significantly across radii (Fig. 4*E*; *F*_(2.737, 57.49)_ = 128.0; *p* < 0.0001), but there were no significant main effects of hormone or drug and no interactions. Additional Sholl-derived measures indicated that basal dendritic volume (Fig. S5*A*), surface area (Fig. S5*B*), length (Fig. S5*C*), and number of nodes (Fig. S5*D*) did not differ by drug, hormone, or their interaction (Data S1).

### E_2_-induced spine remodeling requires proteasome activity, with strongest effects in apical dendrites and more modest parallel effects in basal dendrites

#### CA1 apical dendritic segments

##### Spine density on apical segments

Given the broad effects of E_2_ across the apical arbor, we next examined whether these changes reflected localized alterations within specific dendritic segments. Whereas the Sholl and AUC analyses provide arbor-wide measures of spine distribution, the segment analyses described below provide assessment of spine density, spine subtype density, and morphometric properties within standardized branch-order categories. To this end, two 20 µm tertiary apical segments were selected per neuron for spine type and morphometric analyses (Fig. 5*A*; 300 segments total).

**Figure 5.**
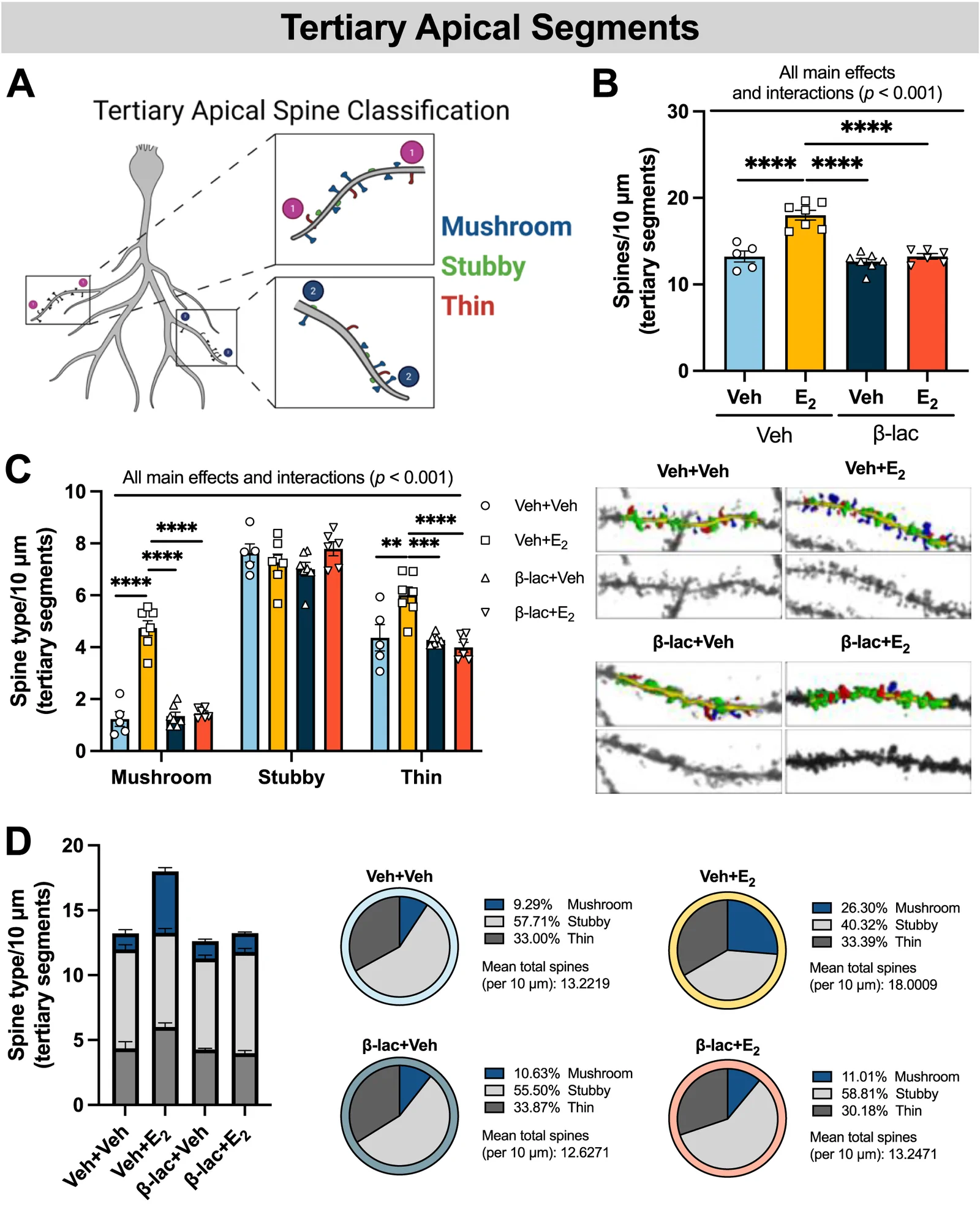
Proteasome activity was required for E_2_-induced, spine-type–specific increases in apical spine density. ***A***, Schematic illustrating selection of tertiary apical dendritic segments. Spines were visually inspected for accuracy and classified as mushroom, stubby, or thin using Neurolucida 360 automated spine detection parameters. ***B***, Veh + E_2_-treated mice exhibited greater total spine density on apical dendrites compared with all other treatment groups (****p < 0.0001), resulting in significant main effects of drug, hormone, and a drug × hormone interaction (all *p* < 0.001). ***C***, Spine-type analysis revealed that Veh + E_2_ treatment selectively increased mushroom and thin spine density relative to all other groups (****p < 0.0001; ***p < 0.001; **p < 0.01). All main effects and interactions were significant (*p* < 0.001). Representative reconstructed apical dendrites are shown to the right, with corresponding raw images below. ***D***, Proportional analysis of spine types illustrates that Veh + E_2_-treated mice exhibited the greatest overall spine density, accompanied by an increase in the proportion of mushroom spines and a corresponding reduction in the proportion of stubby spines. Error bars indicate mean ± SEM.

Spine density on sampled tertiary apical dendritic segments differed significantly by hormone (Fig. 5*B*; *F*_(1,21)_ = 30.40; *p* < 0.0001), drug (*F*_(1,21)_ = 29.83; *p* < 0.0001), and their interaction (*F*_(1,21)_ = 18.04; *p* = 0.0004). These effects were driven by the Veh + E_2_ group, which exhibited ∼36–43% greater spine density than all other treatment groups (all *p* < 0.0001). These findings indicate that E_2_ robustly increased spine density on sampled tertiary apical dendritic segments, and this effect was eliminated by β-lac.

##### Spine-type density on apical segments

To determine whether E_2_ preferentially altered specific spine types, spine subtype density was analyzed by morphological classification (mushroom, stubby, or thin). Spine subtype density differed significantly across spine types (Fig. 5*C*; *F*_(2,63)_ = 366.2, *p* < 0.0001), with stubby spines comprising the predominant spine type on sampled apical dendritic segments. There were significant main effects of drug (*F*_(1,63)_ = 31.87; *p* < 0.0001) and hormone (*F*_(1,63)_ = 32.47; *p* < 0.0001), as well as a significant drug × hormone interaction (*F*_(1,63)_ = 19.27; *p* < 0.0001), indicating that E_2_ broadly increased spine subtype density and that this effect was blocked by β-lac. However, these effects differed by spine subtype, as evidenced by significant spine type × hormone (*F*_(2,63)_ = 9.083; *p* = 0.0003) and spine type × drug interactions (*F*_(2,63)_ = 8.131; *p* = 0.0007). E_2_ selectively increased the mushroom and thin spine density, whereas β-lac reduced the density of these spine subtypes. Accordingly, the spine type × drug × hormone interaction was also significant (*F*_(2,63)_ = 18.00; *p* < 0.0001). Post hoc analyses revealed that the Veh + E_2_ group exhibited ∼225–285% greater mushroom spine density than all other treatment groups (all *p* < 0.0001) and ∼38–50% greater thin spine density than the Veh + Veh (*p* = 0.0052), β-lac + Veh (*p* = 0.0006), and β-lac + E_2_ groups (*p* < 0.0001).

Proportional analyses confirmed that stubby spines constituted the largest fraction across groups, accounting for >50% of spines in all groups but Veh + E_2_. In this group, E_2_ selectively increased the proportion of mushroom spines to >26% (compared with ≤11% for other groups) and reduced the proportion of stubby spines to ∼40% (Fig. 5*D*); these changes were not observed in the β-lac + E_2_ group. Collectively, these findings indicate that E_2_ selectively increased mushroom and thin spine density on tertiary apical dendritic segments and shifted spine subtype composition toward a greater proportion of mushroom spines, effects that were prevented by β-lac.

##### Morphometric analysis of apical spine structure

We next determined whether the E_2_-associated increases in apical spine density were accompanied by changes in spine morphology by analyzing multiple size-related morphometric features from the same tertiary apical segments used for spine density analyses. Significant main effects of spine type were also observed for all morphometric measures (Data S1), consistent with expected differences among morphologically defined spine subtypes.

Average spine volume (cubic micrometers) was significantly influenced by treatment (Fig. 6*A*), with significant main effects of drug (*F*_(1,63)_ = 4.156; *p* = 0.0457) and hormone (*F*_(1,63)_ = 4.346; *p* = 0.0412) and their interaction (*F*_(1,63)_ = 5.958; *p* = 0.0175), indicating that E_2_ increased spine volume and that this effect was blocked by β-lac. Similarly, average spine surface area (square micrometers) was significantly influenced by drug (Fig. 6*B*; *F*_(1,63)_ = 5.834; *p* = 0.0186), hormone (*F*_(1,63)_ = 6.434; *p* = 0.0137), and their interaction (*F*_(1,63)_ = 9.972; *p* = 0.0024). Post hoc analyses revealed that mushroom spines in the Veh + E_2_ group exhibited ∼30–32% greater surface area than those in Veh + Veh (*p* = 0.0458) and β-lac + E_2_ (*p* = 0.0162) groups. Collectively, these findings indicate that E_2_ increased multiple size-related features of apical spines and that β-lac attenuated these structural changes.

**Figure 6.**
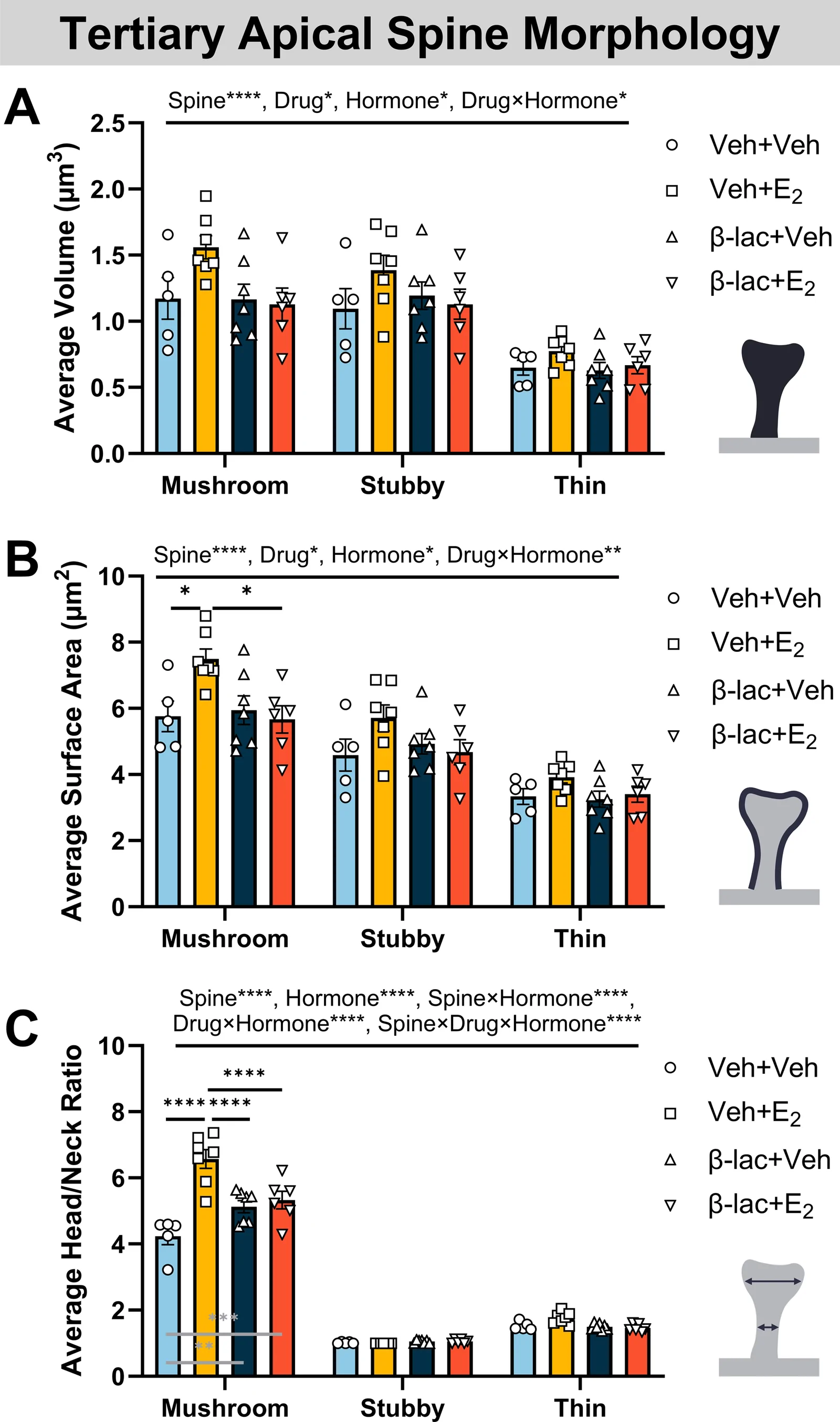
E_2_ enhanced apical spine morphometric features in a spine-type–specific and proteasome-dependent manner. ***A***, Average spine volume differed by spine type, drug, and hormone, with a significant drug × hormone interaction, indicating that E_2_-induced increases in spine volume depend on intact proteasome activity. ***B***, Average spine surface area was significantly influenced by spine type, hormone, and drug, with a significant drug × hormone interaction. Veh + E_2_ treatment selectively increased mushroom spine surface area relative to other treatment groups. ***C***, Average spine head/neck ratio differed significantly by spine type and hormone, with significant spine × hormone, drug × hormone, and three-way spine × drug × hormone interactions. Veh + E_2_ treatment markedly increased mushroom spine head/neck ratio, an effect that was blocked by proteasome inhibition. Statistical symbols reflect main effects and interactions from mixed-effects models with appropriate post hoc comparisons (*p < 0.05; **p < 0.01; ****p < 0.0001). Error bars indicate mean ± SEM.

To further assess spine maturation, we examined spine head/neck ratio, which reflects the relative enlargement of the spine head compared with the neck. Spine head/neck ratio differed significantly by hormone (Fig. 6*C*; *F*_(1,63)_ = 27.63; *p* < 0.0001) and exhibited a significant drug × hormone interaction (*F*_(1,63)_ = 21.33; *p* < 0.0001), indicating that E_2_ increased spine head/neck ratio and that this effect was blocked by β-lac. Significant spine type × hormone (*F*_(2,63)_ = 22.28; *p* < 0.0001) and spine type × drug × hormone interactions (*F*_(2,63)_ = 15.18; *p* < 0.0001) further indicated that these effects differed across spine subtypes, whereas the main effect of drug and spine type × drug interaction were not significant (Data S1). Post hoc analyses revealed that mushroom spines in the Veh + E_2_ group exhibited ∼23–55% greater spine head/neck ratios than all other treatment groups (all *p* < 0.0001).

##### Additional morphometric features of apical spines

Additional morphometric analyses assessed apical spine head extent, neck extent, head diameter, and neck diameter (Fig. S6). Head extent was significantly influenced by drug (Fig. S6*A*; *F*_(1,61)_ = 5.53; *p* = 0.0219), hormone (*F*_(1,61)_ = 19.57; *p* < 0.0001), and their interaction (*F*_(1,61)_ = 25.17; *p* < 0.0001), indicating that E_2_ increased spine head extent and that this effect was blocked by β-lac. These effects were most pronounced in mushroom and thin spines, with the Veh + E_2_ group exhibiting ∼12–21% greater mushroom spine head extent and ∼33–37% greater thin spine head extent than comparison groups (Data S1). Similarly, neck extent was influenced by hormone (Fig. S6*B*; *F*_(1,61)_ = 4.92; *p* = 0.0304) and exhibited a significant drug × hormone interaction (*F*_(1,61)_ = 19.91; *p* < 0.0001), with Veh + E_2_ mice exhibiting ∼28–30% greater thin spine neck extent than Veh + Veh and β-lac + E_2_ mice (Data S1). In contrast, head and neck diameter were unaffected by treatment (Fig. S6*C*,*D*; Data S1).

Collectively, these findings indicate that E_2_ preferentially increased spine extent rather than absolute spine diameter and that these effects were prevented by β-lac. Because the spine head/neck ratio reflects the relative enlargement of the spine head compared with the neck, changes in this metric may occur even in the absence of significant differences in head or neck diameter measured independently.

#### CA1 basal dendritic segments

##### Spine density on basal segments

Given the robust effects of E_2_ on spine density and morphology in tertiary apical dendritic segments, we next examined whether similar effects were observed in secondary basal dendritic segments, where differences in structural plasticity relative to apical dendrites have been reported (Piskorowski and Chevaleyre, 2011; Mihaljevic et al., 2020). Using the same segment-level approach described above, two 20 µm secondary basal dendritic segments were selected per neuron for spine analyses (Fig. 7*A*; 300 basal segments total).

**Figure 7.**
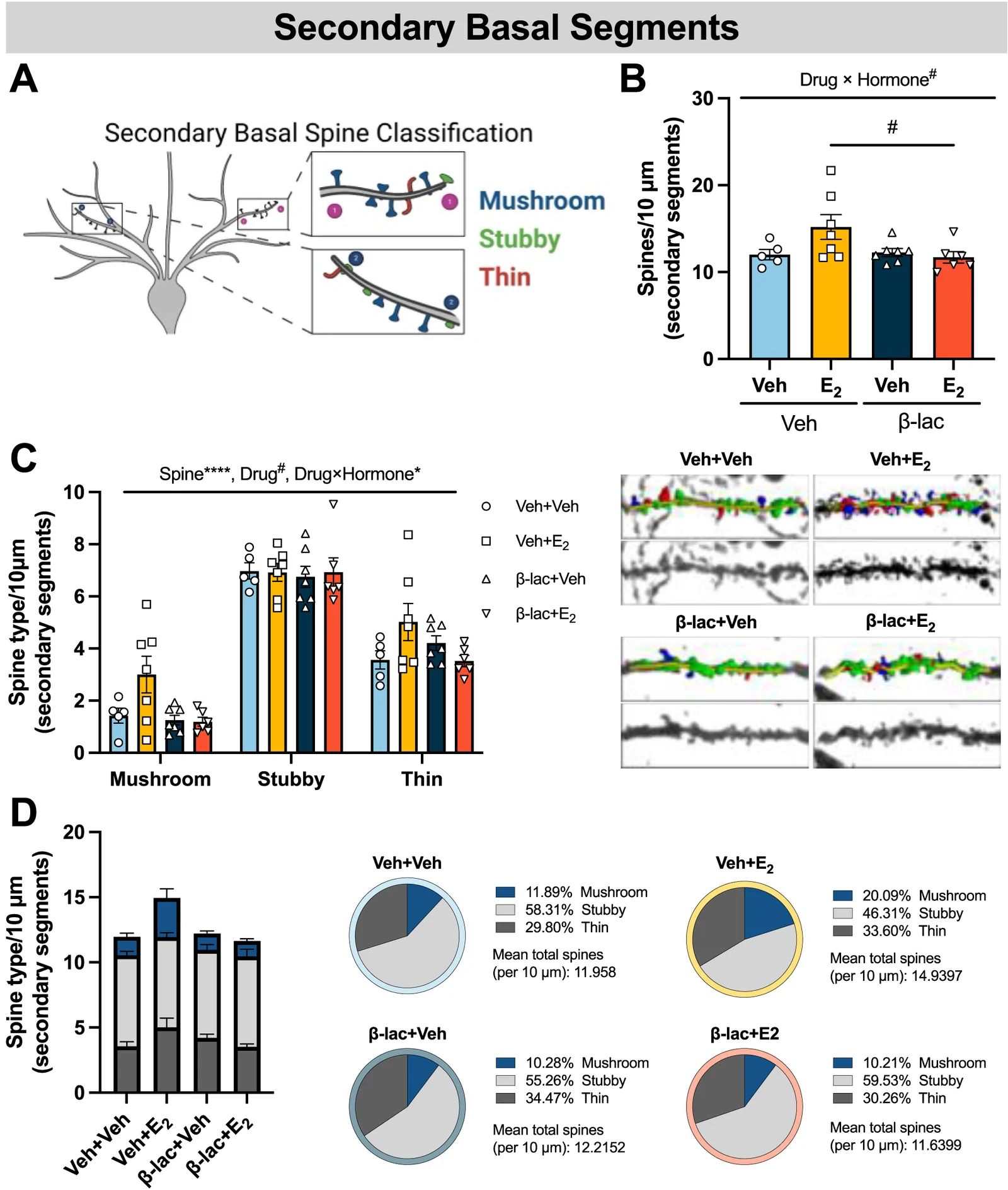
E_2_ produced modest spine-type–specific effects on basal dendrites. ***A***, Schematic illustrating selection of secondary basal dendritic segments and spine categorization. ***B***, Veh + E_2_-treated mice exhibited a trend toward greater total spine density on basal segments compared with β-lac + E_2_-treated mice (^#^*p* < 0.10), contributing to a trend for a drug × hormone interaction. ***C***, Basal spine density differed significantly by spine type (****p < 0.0001) and revealed a significant drug × hormone interaction (*p < 0.05), indicating that E_2_ increased overall spine number on basal dendrites. Although E_2_-treated mice displayed numerically greater mushroom and thin spine densities, post hoc comparisons did not reach statistical significance. Representative reconstructed apical dendrites are shown to the right, with corresponding raw images below. ***D***, Proportional analysis of spine types illustrates that Veh + E_2_-treated mice exhibited the highest overall spine density, accompanied by a relative shift in composition from stubby spines toward mushroom spines. Error bars indicate mean ± SEM.

In contrast to apical dendrites, spine density on sampled secondary basal dendritic segments did not differ significantly by drug or hormone (Fig. 7*B*), although the drug × hormone interaction approached significance (*F*_(1,21)_ = 3.862; *p* = 0.0627). This pattern was driven by a trend for reduced spine density in the β-lac + E_2_ group relative to the Veh + E_2_ group (*p* = 0.0660), suggesting a modest attenuation of E_2_-associated spine increases by proteasome inhibition.

##### Spine-type density on basal segments

As observed in apical dendritic segments, treatment effects in basal segments became more apparent when spine subtypes were examined individually. Spine subtype density differed significantly across spine types (Fig. 7*C*; *F*_(2,63)_ = 136.8; *p* < 0.0001), with stubby spines comprising the most prevalent subtype across segments. There was a main effect of drug (*F*_(1,63)_ = 3.930; *p* = 0.0518) and a significant drug × hormone interaction (*F*_(1,63)_ = 5.370; *p* = 0.0237), indicating that β-lac attenuated E_2_-associated alterations in basal subtype composition. No other interactions or main effects were significant (Data S1).

Proportional analyses demonstrated that stubby spines constituted >55% of spines in all groups except Veh + E_2_, in which the proportion was reduced to ∼46.3%. Conversely, the proportion of mushroom spines increased to ∼20% in the Veh + E_2_ group, representing an approximately twofold increase relative to the β-lac + E_2_ group (Fig. 7*D*). Together, these findings indicate that E_2_-associated changes in basal spine subtype composition were modest but paralleled those observed in apical dendrites, with a relative increase in mushroom spines that was attenuated by β-lac.

##### Morphometric analysis of basal spine structure

Consistent with apical dendritic segments, basal spines also exhibited E_2_- and proteasome-sensitive changes in structural features. Significant main effects of spine type were observed for all basal spine morphometric measures (Data S1).

Average spine volume was significantly influenced by hormone (Fig. 8*A*; *F*_(1,61)_ = 6.125; *p* = 0.0161), indicating that E_2_ increased spine volume overall, and exhibited a trend toward a drug × hormone interaction (*F*_(1,61)_ = 3.169; *p* = 0.0800), suggesting that β-lac attenuated this effect. Similarly, the average spine surface area exhibited a significant main effect of hormone (Fig. 8*B*; *F*_(1,61)_ = 5.967; *p* = 0.0201) and a significant drug × hormone interaction (*F*_(1,61)_ = 4.134; *p* = 0.0464), indicating that β-lac reduced E_2_-associated increases in the spine surface area. Unlike apical dendritic segments, basal segments did not exhibit hormone-dependent changes in spine head/neck ratio (Fig. 8*C*). No additional main effects or interactions were significant (Data S1). Collectively, these findings indicate that β-lac attenuated E_2_-associated increases in multiple basal spine structural measures, with the strongest effects observed for spine surface area and a similar trend for spine volume.

**Figure 8.**
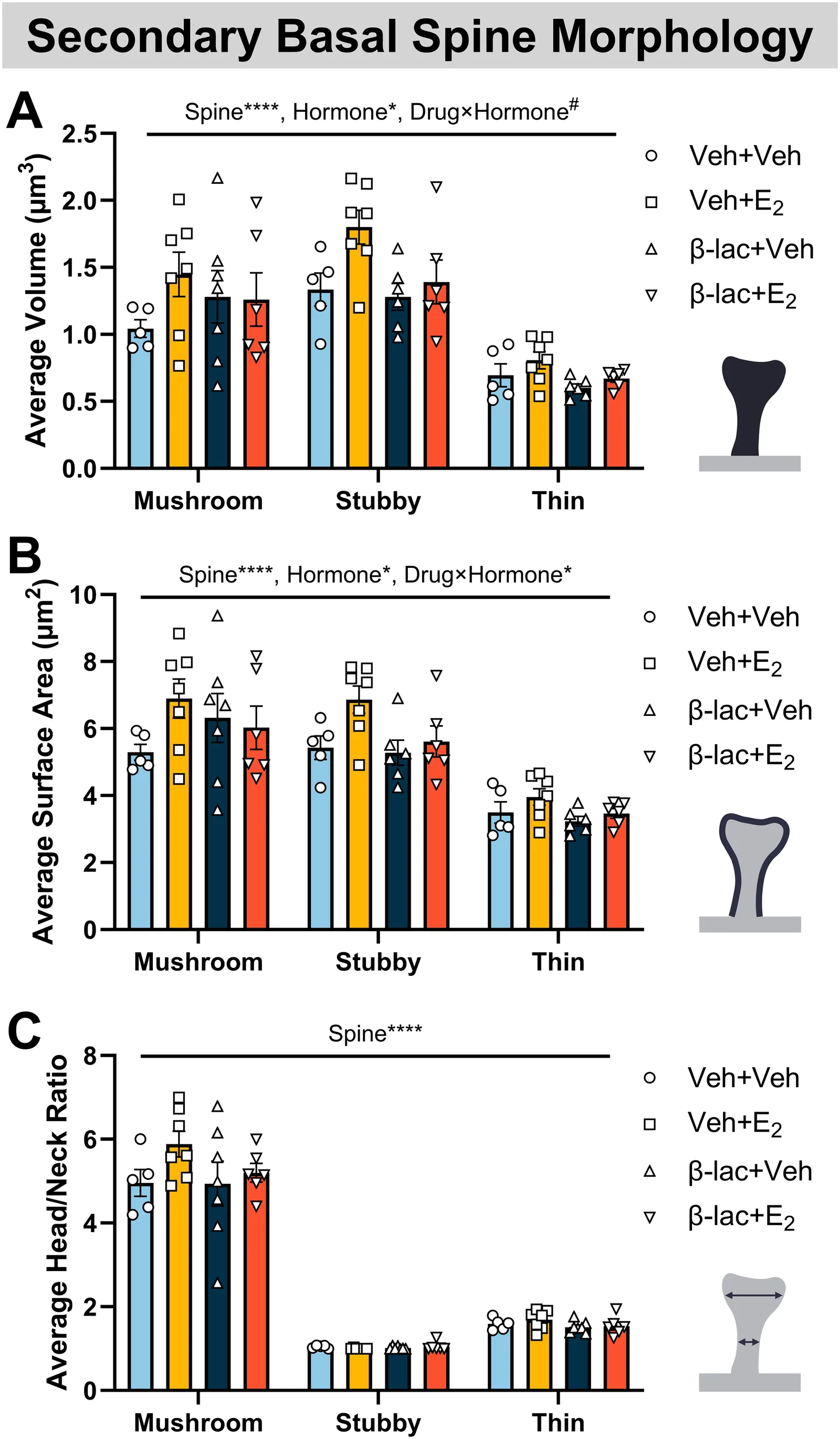
E_2_ drove more modest, proteasome-dependent structural enlargement of spines on basal dendrites. ***A***, Average spine volume differed by spine type and hormone, with a trend toward a drug × hormone interaction, indicating an increase in spine volume by E_2_ that may depend on proteasome activity. ***B***, Average spine surface area varied by spine type and hormone, with a significant drug × hormone interaction, suggesting that E_2_-dependent effects on spine surface area are attenuated by proteasome inhibition. ***C***, Average spine head/neck ratio differed by spine type but was not significantly altered by hormone or drug treatment. Statistical annotations denote main effects and interactions from mixed-effects models (*p < 0.05; ****p < 0.0001; ^#^*p* < 0.10). Error bars indicate mean± SEM.

##### Additional morphometric features of basal spines

Additional morphometric features of basal spines were quantified, including average head extent, neck extent, head diameter, and neck diameter (Fig. S7). Head extent exhibited a significant main effect of hormone [Fig. S7*A*; (*F*_(1,63)_ = 6.306; *p* = 0.0146)], indicating that E_2_ increased head extent overall. Similarly, neck extent exhibited a significant main effect of hormone (Fig. S7*B*; *F*_(1,62)_ = 4.666; *p* = 0.0346) and a trend toward a main effect of drug (*F*_(1,62)_ = 3.704; *p* = 0.0589), suggesting that E_2_ increased neck extent and that β-lac tended to attenuate this effect. No other main effects or interactions were significant for head and neck extent (Data S1). Likewise, no effects of hormone or drug were observed for head diameter (Fig. S7*C*) or neck diameter (Fig. S7*D*; Data S1).

Together, these findings parallel the apical dendritic segment results and indicate that E_2_ preferentially increased the extent, rather than the absolute diameter, of basal spine structures in a proteasome-sensitive manner, albeit with reduced magnitude.

##### Overview and model of apical and basal spine changes

Collectively, these analyses reveal compartment- and scale-specific effects of E_2_ on dendritic spine organization in CA1 (Fig. 9). At the arbor level (Fig. 9*A*), E_2_ produced modest alterations in basal spine distribution but robust increases in spine number throughout the apical dendritic tree. Segment-level analyses (Fig. 9*B*) further demonstrated that the strongest effects of E_2_ occurred on sampled tertiary apical dendritic segments, where E_2_ markedly increased spine density and shifted spine-type composition, whereas sampled secondary basal dendritic segments exhibited smaller and more variable changes. Morphometric analyses further showed that E_2_ increased multiple structural features of apical mushroom spines, including spine volume, surface area, and head/neck ratio. Importantly, many of these E_2_-dependent changes were attenuated or abolished by proteasome inhibition with β-lac, suggesting that proteasome activity contributes to both spine addition and changes in spine structure. Figure 9*C* provides a schematic overview of the effects of E_2_ and β-lac across arbor- and segment-level analyses.

**Figure 9.**
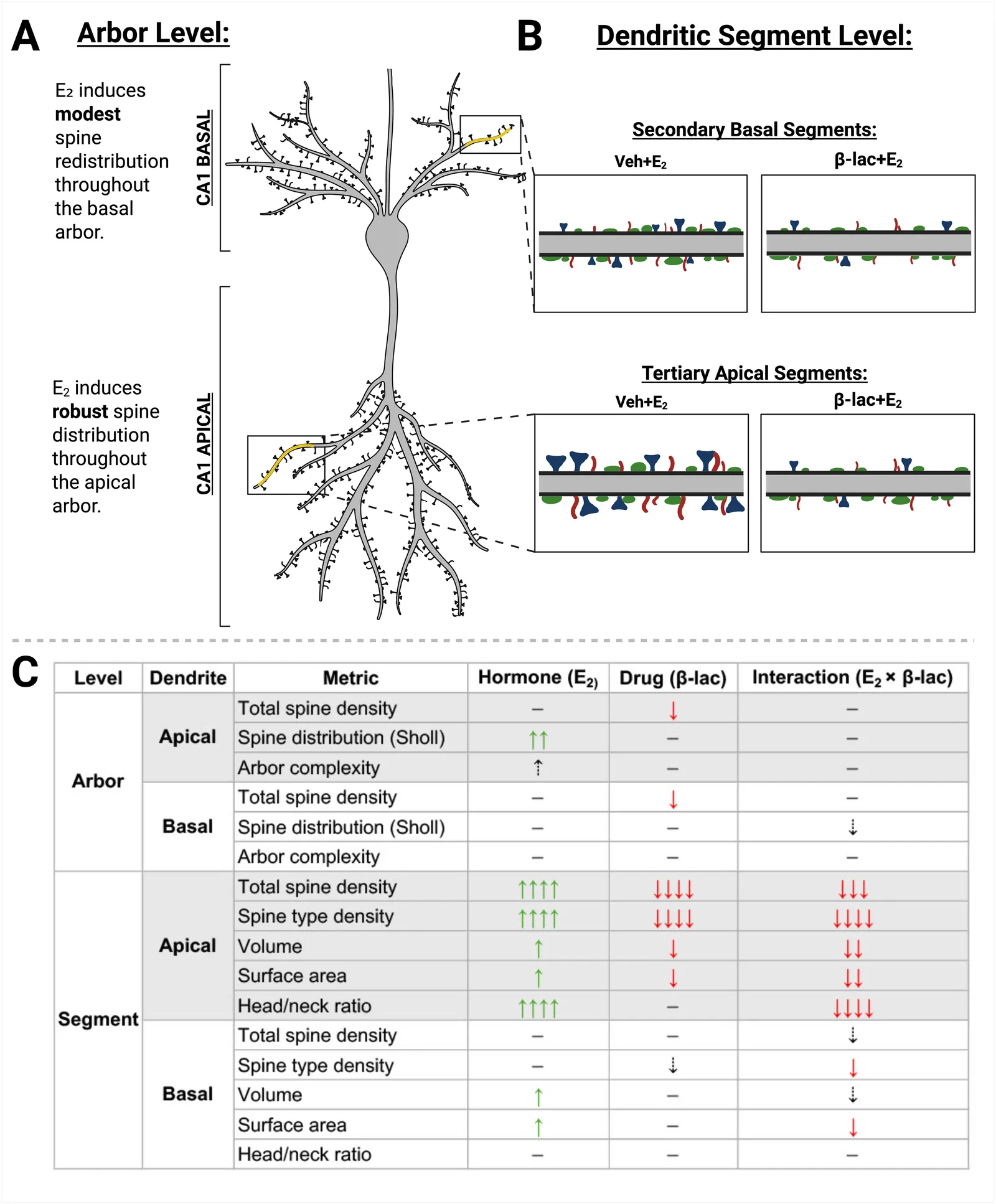
Summary of E_2_- and proteasome-dependent regulation of CA1 dendritic spine remodeling across arbor and segment levels. ***A***, Arbor-level schematic illustrating the overall distribution of dendritic spines across the CA1 apical and basal dendritic trees. E_2_ broadly increased spine number across the apical dendritic arbor, whereas effects across the basal arbor were more modest. ***B***, Segment-level schematic depicting representative apical and basal dendritic segments used for detailed spine analyses. In apical segments, E_2_ robustly increased spine density and promoted structural enlargement of spines, whereas these effects were blocked by proteasome inhibition. In basal segments, E_2_ produced smaller, parallel changes in spine structure and composition that were attenuated by β-lac. Colored shapes illustrate representative spine types (mushroom, stubby, and thin). ***C***, Summary of significant main effects and interactions across dendritic levels, dendrite types, and spine metrics. Green arrows indicate increases associated with E_2_ infusion, red arrows indicate decreases associated with β-lac treatment, arrows in the interaction column indicate significant E_2_  ×  β-lac interactions, and dashes indicate no significant effect. Arrow number denotes statistical significance (*p < 0.05; **p < 0.01; ***p < 0.001; ****p < 0.0001).

## Discussion

Our findings demonstrate that proteasome-dependent protein degradation is required for E_2_ to enhance memory consolidation and remodel CA1 dendritic spines. These findings advance the field in three ways. First, they reveal a vital role for learning-induced proteasome activity in memory consolidation in female mice. Second, they demonstrate that proteasome activity is necessary for E_2_-induced memory enhancement and CA1 dendritic spine remodeling. Third, they provide the first three-dimensional structural approximation of E_2_-induced spine remodeling across CA1 apical and basal dendrites in vivo, revealing dendrite- and spine-type–specific effects not captured by traditional two-dimensional analyses.

In two complementary behavioral experiments, we first established a dose–response profile for post-training DH infusion of the 20S proteasome inhibitor β-lac. Both 16 and 32 ng/µl β-lac blocked object recognition and spatial memory consolidation, consistent with prior reports that 32 ng/µl β-lac impairs object location memory in male mice (Choi et al., 2010) and fear memory in male rats (Jarome et al., 2011; Reis et al., 2013). Here, we extend this work by demonstrating a similar dependence on proteasome activity in ovariectomized female mice. Importantly, combining a behaviorally subthreshold 8 ng/µl dose of β-lac with E_2_ prevented E_2_-induced memory enhancement, providing the first direct evidence that proteasome-dependent protein degradation is required for estrogenic facilitation of hippocampus-dependent memory. Together with evidence that protein synthesis is also required for E_2_-induced memory consolidation (Fortress et al., 2013; Tuscher et al., 2016), these findings suggest that coordinated regulation of protein synthesis and degradation may support E_2_-dependent plasticity. Although sex differences in proteasome engagement have been reported during object (Beamish et al., 2022) and fear memory consolidation (Martin et al., 2021), the contribution of endogenous sex steroid fluctuations to these differences remains unclear.

Although E_2_ is known to increase hippocampal dendritic spine density, our findings provide a three-dimensional structural approximation of how E_2_ remodels dendritic spines across both apical and basal CA1 dendrites in vivo. By combining Golgi staining with automated spine detection, we achieved large-scale, morphometric analysis and detected ∼32% more spines than in our previous manual two-dimensional analyses (Tuscher et al., 2016; Kim et al., 2019; Taxier et al., 2022), likely due to improved visualization of spines surrounding the dendritic shaft. The improved sensitivity revealed that E_2_ engages proteasome-dependent protein degradation to coordinate changes in spine-type composition and morphology.

Arbor-wide analyses showed that E_2_ increased spine number throughout the apical dendritic tree, whereas basal changes were smaller and more variable. This widespread apical remodeling is consistent with previous reports of E_2_-induced spine formation across dendritic segments in the primate prefrontal cortex (Hao et al., 2006). Consistent with these findings, spine density on apical dendritic segments increased by ∼36%, closely matching the ∼30% increase previously reported (Tuscher et al., 2016), whereas basal dendritic segments showed a similar but more variable trend.

Beyond increases in spine density and distribution, E_2_ altered spine-type composition, preferentially increasing mushroom and thin spines on apical dendrites, consistent with previous reports of E_2_-induced increases in mushroom (Gould et al., 1990; Li et al., 2004; McLaughlin et al., 2008, 2010; Kim et al., 2019) and thin spines (Murakami et al., 2006; Mukai et al., 2007; Srivastava et al., 2008, 2010). Because mushroom spines are associated with stable, stronger excitatory synapses, whereas thin spines are more structurally plastic (Harris et al., 1992; Bourne and Harris, 2007), these findings suggest that E_2_ may both stabilize existing excitatory contacts and expand the population of spines available for further remodeling. Importantly, these coordinated changes in spine subtype composition require proteasome activity.

Morphometric analyses further revealed that E_2_ increased mushroom spine volume, surface area, and head/neck ratio on apical dendritic segments, structural features associated with larger postsynaptic densities and enhanced synaptic strength (Harris and Stevens, 1989; Matsuzaki et al., 2001; Arellano et al., 2007). E_2_ also increased the head extent of mushroom and thin spines and neck extent of thin spines, suggesting coordinated enlargement of both mature and plastic spine populations. These effects were blocked by proteasome inhibition, demonstrating that proteasome activity is required for both E_2_-induced spine formation and structural maturation. In contrast, basal dendrites exhibited weaker morphometric responses and lacked comparable mushroom spine enlargement, consistent with reports that apical dendrites are more sensitive to hormone-dependent remodeling (Gould et al., 1990; Woolley et al., 1990a, b, 1994; Murakami et al., 2006; McLaughlin et al., 2008, 2010; Brocca et al., 2013). The greater apical sensitivity may reflect differences in afferent input, as apical dendrites integrate long-range excitatory and modulatory projections, and prior work suggests that E_2_-induced CA1 remodeling is influenced by subcortical afferent systems (Leranth et al., 2000), suggesting that compartment-specific circuitry may contribute to greater apical sensitivity to estrogenic remodeling.

Importantly, proteasome inhibition blocked many of the E_2_-induced changes in spine density, spine-type composition, and structural morphology without reducing spine density below vehicle levels, suggesting that proteasome activity is required for E_2_-driven dendritic spine remodeling. The rapid onset of these structural changes (within 2 h) suggests a selective requirement for protein degradation during the consolidation window. In hippocampal neurons, proteasomes rapidly translocate into dendritic spines following synaptic stimulation, enabling local degradation of proteins that constrain AMPA receptor trafficking and postsynaptic expansion (Bingol and Schuman, 2004, 2006; Bingol et al., 2010; Ehlers, 2003; Pak and Sheng, 2003; Jarome et al., 2011). E_2_ may similarly promote activity-dependent recruitment of proteasomes to dendritic spines to facilitate spine growth and remodeling. Consistent with this possibility, E_2_ promotes ubiquitination of GluA1-containing AMPA receptors in the hippocampus (Briz et al., 2015), as well as ERα-dependent ubiquitination of Cav1.2 channels in cortical neurons (Lai et al., 2019), suggesting that E_2_ signaling can directly engage the UPS to regulate synaptic protein composition.

Several limitations constrain mechanistic interpretation of our data. First, the mechanisms through which E_2_ requires proteasome activity to enhance memory consolidation and dendritic spine remodeling remain unclear. Because β-lac was infused into the DH, proteasome inhibition likely affected multiple hippocampal cell types and signaling pathways beyond CA1 pyramidal neurons. Thus, the spine changes likely represent one consequence of disrupted UPS signaling rather than the sole mechanism underlying E_2_-induced memory enhancement. Accordingly, the structural changes observed here should be viewed as associated with, rather than directly responsible for, memory enhancement.

We also cannot determine whether E_2_ actively upregulates proteasomal degradation or instead requires intact proteostatic balance to exert its effects. Although pharmacological inhibition allowed us to temporally restrict proteasome disruption to the consolidation window, β-lac may indirectly impair E_2_-induced memory enhancement by promoting broader accumulation of ubiquitinated proteins rather than selectively preventing degradation of E_2_-relevant substrates. Nevertheless, because spines were analyzed 2 h after a single infusion, widespread depletion of the ubiquitin pool is unlikely in this timeframe, although it cannot be entirely excluded. More broadly, because β-lac did not significantly reduce proteasome activity in our in vivo assay, we cannot exclude the possibility that additional or unintended effects of β-lac contributed to its interaction with E_2_.

An additional limitation is that dendritic spine morphology was used as a structural proxy for synaptic remodeling. Although spine size and morphology frequently correlate with synaptic protein composition and synaptic strength (Harris et al., 1992; Bourne and Harris, 2007), dendritic spines do not necessarily represent functional synapses. Therefore, the present findings should be interpreted as evidence of structural remodeling that may be associated with altered synaptic function, rather than direct evidence of synaptic plasticity. Future studies will be necessary to determine whether the structural remodeling observed here is accompanied by corresponding changes in synaptic function, including alterations in synaptic transmission and synaptic protein composition.

Dendritic spine morphometry was also performed using conventional bright-field microscopy, which is ultimately constrained by the diffraction limit of light microscopy, particularly for submicron spine features. Although other methods (e.g., electron microscopy, Lucifer yellow impregnation) offer greater morphological accuracy for resolving the features of objects as small as dendritic spines, such methods are challenging or impossible to use in an in vivo design such as this (Walker et al., 2022). Accordingly, the Golgi-based morphometric measurements reported here should be interpreted within the resolution limits of the staining method and imaging system. Nevertheless, all tissue was processed, imaged, reconstructed, and analyzed using identical acquisition parameters, thus permitting comparisons among E_2_ and β-lac treatment groups within these methodological constraints.

Lastly, although E_2_ increased spine density and enlargement, it remains unclear whether these effects reflect de novo spine formation, maturation of existing spines, or stabilization of transient spines or a combination of these processes. Prior live-imaging studies suggest that E_2_ preferentially promotes immature thin spines (Srivastava et al., 2008; Mendez et al., 2011; Wang et al., 2018), and longitudinal in vivo imaging has demonstrated dynamic CA1 dendritic spine remodeling across estrous cycle (Wolcott et al., 2025); however, methodological differences preclude direct comparison with the present findings. Future longitudinal imaging studies will be needed to resolve these possibilities.

In conclusion, our findings identify protein degradation as a previously underappreciated mechanism supporting E_2_-dependent synaptic plasticity and memory consolidation. By linking E_2_ signaling to proteasome-dependent spine remodeling, this work provides a framework for understanding how hormonal signaling shapes synaptic structure and cognitive function.
